# Neurological and behavioral abnormalities, ventricular dilatation, altered cellular functions, inflammation, and neuronal injury in brains of mice due to common, persistent, parasitic infection

**DOI:** 10.1186/1742-2094-5-48

**Published:** 2008-10-23

**Authors:** Gretchen Hermes, James W Ajioka, Krystyna A Kelly, Ernest Mui, Fiona Roberts, Kristen Kasza, Thomas Mayr, Michael J Kirisits, Robert Wollmann, David JP Ferguson, Craig W Roberts, Jong-Hee Hwang, Toria Trendler, Richard P Kennan, Yasuhiro Suzuki, Catherine Reardon, William F Hickey, Lieping Chen, Rima McLeod

**Affiliations:** 1 Department of Ophthalmology, University of Chicago, Chicago, IL, USA; 2 Committee on Immunology, University of Chicago, Chicago, IL, USA; 3 Department of Pathology, Cambridge University, Cambridge, UK; 4 University Department of Pathology, Western Infirmary, Glasgow, UK; 5 Department of Health Studies, The University of Chicago, Chicago, IL, USA; 6 Department of Pathology, University of Chicago, Chicago, IL, USA; 7 Nuffield Department of Pathology, Oxford University, Oxford, UK; 8 Department of Immunology, Strathclyde Institute of Pharmacy and Biomedical Sciences, University of Strathclyde, Glasgow, UK; 9 Albert Einstein College of Medicine, Bronx, NY, USA; 10 Department of Biomedical Sciences and Pathobiology, Virginia-Maryland Regional College of Veterinary Medicine, Virginia Polytechnic Institute & State University, Blacksburg, VA, USA; 11 Department of Pathology, Dartmouth University School of Medicine, Hanover, NH, USA; 12 Department of Pathology, Johns Hopkins School of Medicine, Baltimore, Maryland, USA; 13 Department Pediatrics (Infectious Diseases); Committees on Molecular Medicine, Genetics, and The College, University of Chicago, Chicago, IL, USA

## Abstract

**Background:**

Worldwide, approximately two billion people are chronically infected with *Toxoplasma gondii *with largely unknown consequences.

**Methods:**

To better understand long-term effects and pathogenesis of this common, persistent brain infection, mice were infected at a time in human years equivalent to early to mid adulthood and studied 5–12 months later. Appearance, behavior, neurologic function and brain MRIs were studied. Additional analyses of pathogenesis included: correlation of brain weight and neurologic findings; histopathology focusing on brain regions; full genome microarrays; immunohistochemistry characterizing inflammatory cells; determination of presence of tachyzoites and bradyzoites; electron microscopy; and study of markers of inflammation in serum. Histopathology in genetically resistant mice and cytokine and NRAMP knockout mice, effects of inoculation of isolated parasites, and treatment with sulfadiazine or αPD1 ligand were studied.

**Results:**

Twelve months after infection, a time equivalent to middle to early elderly ages, mice had behavioral and neurological deficits, and brain MRIs showed mild to moderate ventricular dilatation. Lower brain weight correlated with greater magnitude of neurologic abnormalities and inflammation. Full genome microarrays of brains reflected inflammation causing neuronal damage (Gfap), effects on host cell protein processing (ubiquitin ligase), synapse remodeling (Complement 1q), and also increased expression of PD-1L (a ligand that allows persistent *LCMV *brain infection) and CD 36 (a fatty acid translocase and oxidized LDL receptor that mediates innate immune response to beta amyloid which is associated with pro-inflammation in Alzheimer's disease). Immunostaining detected no inflammation around intra-neuronal cysts, practically no free tachyzoites, and only rare bradyzoites. Nonetheless, there were perivascular, leptomeningeal inflammatory cells, particularly contiguous to the aqueduct of Sylvius and hippocampus, CD4+ and CD8+ T cells, and activated microglia in perivascular areas and brain parenchyma. Genetically resistant, chronically infected mice had substantially less inflammation.

**Conclusion:**

In outbred mice, chronic, adult acquired *T. gondii *infection causes neurologic and behavioral abnormalities secondary to inflammation and loss of brain parenchyma. Perivascular inflammation is prominent particularly contiguous to the aqueduct of Sylvius and hippocampus. Even resistant mice have perivascular inflammation. This mouse model of chronic *T. gondii *infection raises questions of whether persistence of this parasite in brain can cause inflammation or neurodegeneration in genetically susceptible hosts.

## Background

The protozoan parasite *Toxoplasma gondii *remains as a chronic, cryptic, latent brain infection throughout the life of the host [1]. Understanding the effects of chronic *T. gondii *infection is particularly important because this parasite chronically infects 30–50% of the human population worldwide [1]. There are a variety of reports that suggest that chronic *Toxoplasma *infection may alter human behaviors, cognitive functions, and cause cryptogenic epilepsy, headaches, and onset of schizophrenia [e.g., [2-4]]. In these studies, investigators have noted increased seroprevalence for past *T. gondii *infection or increased magnitude of antibody titers specific for *T. gondii *in sera of persons with these medical problems [5]. Limitations of some of these studies have been discussed [6]. None definitively prove a cause and effect relationship [6].

At the same time, there have been a variety of often contradictory reports of isolated and specific neurological abnormalities in chronically infected, conventionally housed (and thus possibly concomitantly infected), mice or rats [7-15]. Some of these studies of prolonged *T. gondii *infection in conventionally housed mice simply report general neurological and behavioral abnormalities [7,8], and others suggest there is a specific survival benefit to *T. gondii*, by producing effects such as lack of fear and inability to smell cat urine [9-16]. These are behaviors that could imply that parasites specifically manipulate rodent brains to render rodents more susceptible to capture by a definitive feline host and thus to greater propagation by highly infectious sporulated oocysts formed in and excreted only by cats [12,15].

Earlier investigations of behavioral and neurologic findings have not maintained and documented a specific pathogen free (SPF) status of the mice or rats studied [7-16]. *T. gondii *infection modulates both immune responses and outcomes of many concomitant infections and tumors. The outcome of *T. gondii *infection has been reported to be modulated substantially by presence of either prior infections or concomitant infections [17-32]. Earlier studies have attributed a variety of different findings to *T. gondii *infection (e.g. congenital malformations) when the findings were actually due to a virus contaminating the *T. gondii *cultures [18,19]. In non-SPF mice, behavioral changes ascribed to *T. gondii *infection may have been due to the parasite itself, to a concomitant infection that causes neurologic damage, to a concomitant infection modulating the pathology that *T. gondii *causes, to *T. gondii *infection modulating the pathology a concomitant infection causes, or to some combination of these.

In addition to the confounding factors of concomitant infection, this parasite infects many animals in which genetics of the host (between and within species) and parasite and their interactions determine different outcomes of the primary acute acquired, reactivated chronic, and congenital infection [33-41]. It has not been recognized previously, however, that host genetics effect outcomes of postnatally acquired infections with *T. gondii *that are present for > 5 months or that resistant strains of mice [36] chronically infected for prolonged times have neuropathology.

There also are studies documenting that *T. gondii *modulates a variety of functions in cultured human host cells (e.g., monocytes, fibroblasts, and retinal cells) [42-47] and that this parasite increases expression of Human Endogenous Retroviruses (HERVs) in a human neuronal cell line [48]. The latter finding is similar to findings reported in brain tissue from persons with schizophrenia [49]. However, effects of chronic *T. gondii *infection on transcriptomes and proteomes of brain or individual neuronal cells from different regions of brain have not been reported.

Furthermore, bradyzoites in cysts have been noted to fill neuronal cells that nonetheless form synapses [50,51]. Recently, environmental conditions have been shown to modulate neurotransmission at individual synapses [52], but whether, and if so how *T. gondii *infection of neuronal cells might alter neurotransmission remains to be determined.

Cyst burden or neuropathology has been reported in some strains of mice infected for a month or two [15,36,53]. However, one to two months following infection would still be considered an acute or subacute infection in humans [1,54].

None of the following have been previously reported using mice infected for longer than 5 months: brain weight and size, MRI studies, histopathology in specific pathogen free mice infected with *T. gondii*, histopathologic examinations at well-standardized times many months after initiation of infection, pathology in various regions of the brain, correlation of histopathology with multiple neurological/behavioral tests, and whether pathologic changes observed could be due to concomitant exposure to brain tissue initiating an autoimmune response to brain rather than the parasite itself. Pathogenesis of neuropathologic changes and their distribution has not been clarified for either acutely or chronically infected mice.

The purpose of the studies herein is to begin to fill in these current gaps in the literature and to provide a foundation upon which questions regarding the effects of chronic *T. gondii *infection, such as whether chronic infection could play a role as a co-factor in some behavioral, neurological or other neurodegenerative diseases in humans [55-61], can be addressed.

## Methods

### Descriptions of mice and T. gondii infection of mice for behavioral and neurologic assessments, MRIs, microarrays, and histopathology

#### Swiss Webster mice

Female Swiss Webster (SW) mice were bred in our (RM) laboratory and were derived from breeding pairs originally obtained from Harlan Laboratories (Indianapolis, IN). These outbred females mice were between five and seven weeks of age at the start of the experiments. Animals were housed 5 or less/cage using an individually ventilated rack caging system from Allentown Caging Equipment Company (Allentown, NJ). The Micro Barrier System cages (Allentown Caging Equipment Company) each had an internal area of 67 square inches (7.5 × 11.5 × 5 inches). The colony room was maintained at 22°C with a 12–12 hour light/dark cycle (lights on at 600 h CST). All animals were handled at least once/week for 10–15 seconds. Food and water were available continuously and the care and use of mice was in accordance with AALAC and institutional guidelines. The colony was documented to be free of a large number of viral, bacterial, and parasitic agents [62]. Specifically, testing for the pathogens listed below was performed monthly throughout the studies of SW mice and all test results throughout the studies were negative.

#### Viral pathogens

Mouse Hepatitis Virus (MHV); Coronavirus; Lymphocytic Choriomeningitis Virus (LCMV); Minute Virus of Mice (MVM); Mouse Encephalomyelitis Virus (GDVII); Reovirus-2; Enteric Disease of Infant Mice (EDIM); K virus; Pneumonia Virus of Mice (PVM); Ectromelia virus; Sendai virus; Polyoma virus; Mouse Cytomegalovirus (MCMV); Mouse Adenovirus; Hantaan Virus Mouse Thymic Virus (MTV)

#### Bacterial pathogens

Mycoplasma pulmonis; Salmonella spp.; Citrobacer rodentium; Clostridium piliforme; Cilia Associated Respiratory (CAR) Bacillus

#### Parasitic pathogens

Endoparasites; Pinworms (Syphaciasp, Aspiculuris tetraptera); Tapeworms (Hymenolepis sp.); Protozoa (Giardia muris, Encephalitozoon cuniculi); Ectoparasites; Mites (Myobia musculi, Myocoptes musculinus Radfordia affinis, Psoregates simplex)

Outbred mice used for electron microscopic studies were maintained in non-SPF conditions in Glasgow, Scotland (DF).

**DBA mice **used for the MRI (RK, J-HH) study were SPF when obtained, and both controls and *T. gondii *infected mice were housed in identical conditions in the same colony at Albert Einstein College of Medicine where the MRI facility available for this work was located. Age and strain controls were matched for the MRI studies as for all other studies in this manuscript.

### Infection with T. gondii

For the studies described herein, *Toxoplasma *cysts of the avirulent Me49 strain were obtained from the brains of female Swiss Webster mice that had been infected intraperitoneally (i.p.) between 6 and 16 weeks earlier. Brains from the infected mice were homogenized in sterile saline and an aliquot was used to count cysts. The suspension was diluted in PBS pH 7.4 and 100 Me49 cysts were administered i.p. into mice.

### Experimental design/cohorts studied

A pilot experiment, to characterize and develop all the behavioral and neurologic assessment measures, was performed with mice infected with *T. gondii *when they were 1.5 or 7 months of age, and control mice. Behavioral and neurologic evaluations were performed when they were ~12 months of age.

Then, an initial cohort of mice was studied systematically. This cohort included 6 uninfected female SW, SPF control mice and 10 female SW, SPF mice infected (as described above) with *T. gondii *at ~1.5 months of age. The control mice were age-matched to the infected mice and were bred, born, and housed in the same colony. This cohort was observed and behavioral and neurologic phenotypes documented using all the behavioral and neurologic assessments described in the methods section 11 to 16 months post infection with *T. gondii*.

In order to determine whether the results found in the pilot and initial cohorts could be generalized, a subset of the behavioral and neurologic measures were studied in experiments in two additional studies continued over time (called "kinetic replicate studies"). The two replicate cohorts each included 5 control mice and 5 mice that were infected at 7 months of age and a subset of assessments were conducted monthly from 3 to 5 months post infection.

### Descriptions of mice for histopathologic studies

Additional separate histopathologic studies and studies to better understand pathogenic mechanisms (described below) were performed with 1) SPF, female SW mice infected with Me49 *T. gondii *as above or that were uninfected controls; 2) DBA, female SPF mice infected with Me49 parasite without brain, and matched controls; 3) the DBA mice for which MRIs were performed; 4) genetically resistant [36] SPF, female BALB/c mice that were either uninfected controls or infected with Me49 *T. gondii *as described above; 5) IL4 or IL6 or IL13 or *NRAMP *knockout mice [63-66] that were infected with the Beverly strain of *T. gondii *or uninfected controls.

### Behavioral assessments

Assays for physical appearance and autonomic characteristics, exploratory behavior, neuromuscular function and sensorimotor function (described below) were developed in a pilot study before being used to assess the initial and additional cohorts described above. To conduct these assays, mice were transferred from group housing to individual home cages. After a thirty-minute period of habituation, animals were assessed using a series of well-established ordinal scales [67,68] to categorize a broad array of health and behavioral features as well as indices of motor/neural function.

All measures were determined for the pilot and initial cohort. Illness behavior, general appearance, activity, gait, exploratory behavior, and sensorimotor function were assessed in the additional, kinetic studies.

### Physical appearance and autonomic characteristics

#### Illness behavior

Sick rodents often show characteristic hunched posture, ruffled fur and a reluctance to move. Illness related behaviors and general appearance were assessed using the following scales:

#### Hunching

Healthy rodents often display a hunching posture during periods of quiescence. Hunching was scored as (0) absence of hunching; (1) presence of hunching.

#### Piloerection

Piloerection is a measure of autonomic instability and a sign of distress. It was scored as (0) None; (2) Slight; (4) Moderate; (6) Marked; (8) Extreme. This was analyzed as absent = 0 or present = 1.

#### Grooming/Fur

Self-grooming and social grooming are ongoing behaviors in mice and occur sporadically during the day. Sick or distressed animals often stop grooming, and their coats can appear ruffled, yellow, gray and/or sticky. Grooming was scored as (0) good; (1) sub-normal; (2) poor; or (3) very poor. This was also analyzed as good = 0 or as not good = 1.

#### Palpebral closure

Palpebral closure is a measure of autonomic instability and a sign of distress. It was scored as (0) normal, eyes wide open; (2) 1/4 closed; (4) 1/2 closed; (6) 3/4 closed; (8) completely closed. This was analyzed as normal = 0 or as abnormal = 1.

Lacrimation

This was analyzed as absent = 0 or present = 1.

#### Salivation

This was scored as (0) None; (2) Very slightly wet; (4) Wet zone 1/4 sub-maxilla area; (6) Wet zone 1/2 sub-maxilla area; (8) Wet zone entire sub-maxilla area. This was analyzed as normal = 0 or abnormal = 1.

#### Urination/Defecation

Thought to be a measure of autonomic nervous system tone, urination and/or defecation during brief periods of handling were recorded.

#### Tail wounds

Presence of tail wounds or of severe shortening of tails, highly atypical findings in rodents and salient in this infected phenotype. This was analyzed as present = 1 or absent = 0.

#### Body position/posture

Observations of vertical posture were made and body position was recorded as a still photograph of each animal. Measurements of degree of body leaning were made using the recorded image of each animal. This was analyzed as normal = 0 or abnormal body position (leaning) = 1.

#### Body weight

Animals were weighed, often a sufficient measure of general health, at the outset of the experiment and when behavioral testing was complete.

#### Tremor

Tremors were scored on the following scale (0) None; (2) Slight fine body tremor (1.5 mm); (4) Moderate coarse (3 mm) with slight impairment in locomotion; (6) Marked coarse (4.5 mm) with moderate marked impairment; (8) Extremely coarse (6 mm) with locomotion impossible. Tremor was analyzed as present = 1 or absent = 0.

#### Activity

Mice were transferred from group housing to individual home housing boxes. After a thirty-minute period of habituation, animals were assessed using a series of well-established ordinal scales to categorize a broad array of health and behavioral features as well as indices of motor/neural function [67,68].

#### Gait

This was scored as (0) normal gait and exploratory pattern; (1–2) mild incoordination of gait; (3–7) moderate incoordination; and (8–10) severe disability in moving one or both hind legs with the higher number the most severe. This was analyzed as normal = 0 or abnormal = 1 in the more extensive study.

#### Transfer arousal

Level of transfer arousal in the individual home cage was assessed using a nine point scale: (0) Coma; (1) Marked dulled; (2) Moderately dull; (3) Sub-alert; (4) Alert, active; (5) Hyperalert; (6) Slightly excited; (7) Moderately excited ("hypomania"); (8) Extremely Excited ('hypermanic'). Alert, active behavior was considered normal = 0 and other indicated behaviors as abnormal = 1.

#### Spatial locomotion

Level of spatial locomotion in the test cage was noted as (0) none; (1) slow; (2) active. This was analyzed as active = 0 or none/slow = 1.

#### Stereotyped behavior

Invariant and perseverating motor patterns included (a) Wild running; (b) Constant circling; (c) Excessive grooming; (d) Head bobbing; (e) Freezing. This was analyzed as absence of stereotyped behavior = 0 and presence of any stereotyped behavior = 1.

### Exploratory behavior

#### Open-field testing

Mice were placed in a stainless steel field 40 cm × 40 cm × 40 cm, consisting of 4 sides joined to form a square. The box rested on a plastic platform on the floor; the floor of the experimental field was covered with a single layer of dark cage paper often used in animal cages. The only object present in the field was a stainless steel bowl into which the animal was gently placed at the beginning of testing. In-room observers recorded latency (sec) to move from the animal's initial position, and the number of sniffs, rears, freezes and visits to the center of the field. The open field study was a five minute timed exposure to the open field.

### Neuromuscular function

#### Grip strength

Each mouse was placed on the top of a standard wire cage lid, the lid was shaken slightly allowing the mouse to grip the wires; the lid was then gradually turned upside down. The upside down lid was held approximately 20 cm above soft padding, thus the mouse could not climb down but was safe in the event of a fall. Investigators used a stopwatch to measure latency to fall off the wire lid.

#### Vertical pole test

A metal pole, approximately 2 cm in diameter and 40 cm long and wrapped with tape for improved traction was autoclaved prior to testing. Each mouse was placed in the center of the pole, which was held in a horizontal position. The pole was then gradually lifted to a vertical position. Deficits in motor coordination and balance were detected when mice lost balance on the pole, usually before the pole reached a 45° angle (See Movie, Additional file 1, online).

### Sensorimotor function

#### Pain insensitivity

An autoclaved paper clip was placed at the base of the animal's tail to test pain sensitivity and sensorimotor integration. Latency to remove and/or attempt to remove the paper clip was timed. Maximum trial length was thirty seconds in the first experiment and sixty seconds in the next two experiments.

### MRI

T1 weighted and T2 weighted magnetic resonance imaging was performed on a 9.4 Tesla imaging system (Varian console) at the Albert Einstein School of Medicine. The T1 weighted gradient echo images were acquired to investigate gray and white matter abnormalities using a multi-slice inversion recovery gradient echo sequence. The T2 weighted images were acquired to highlight fluid using a spin echo sequence with the following imaging parameters: TR = 3 s, TE = 60 msec. Image resolution was 110 × 110 microns in plane and 800 microns through plane with 16 slices spanning from rhinal fissure to cerebellum. Slice thickness was 1 mm. Six infected and 3 uninfected age-matched control mice were imaged at 1 year of age. Five of the infected mice had been infected for 8 months, the pilot mouse had been infected for 1 year when imaged. Ventricular size was measured using the histogram feature in Adobe Photoshop to calculate the number of pixels in a given area. The area (in pixels) of the ventricles was divided by the area (in pixels) of the entire brain in that frame for each mouse. The resulting ratio for each mouse was compared using a two-tailed, two-sample t-test.

### Gene expression and statistical analysis of microarrays

The RNA from the brains of three SPF, SW mice infected with *T. gondii *for a minimum of one year and three uninfected SPF, SW control mice bred and housed from birth in the same colony were extracted using trizol (Invitrogen, Carlsbad, CA). Brain was homogenized in saline (1 mL). 1 ml trizol was added to each sample of the cell pellets. This was incubated at room temperature for 5 minutes. Two hundred microliters of chloroform was added to each sample. This was shaken vigorously for 15 seconds and then incubated for 2 minutes at room temperature. This was then centrifuged at 12000 g for 15 minutes at 4 degrees C. The aqueous phase was transferred to a fresh tube and RNA was precipitated with .5 ml of isopropanol per sample. Samples were incubated at room temperature for 10 minutes and then centrifuged again at 12,000 g for 10 minutes at 4 degrees C. The gel like pellet of RNA was washed with 1 ml of 75% ethanol per sample. The sample was vortexed and re-centrifuged at 7500 g for 5 minutes at 4 degrees C. The ethanol was removed and the RNA pellet was air-dried. The sample was resuspended in RNA free water and quantitated using a spectrophotometer (260 lamba). The extinction coefficient aliquots of 40 μg (260/280 was less than 1.6) were utilized for hybridization experiments.

For hybridization experiments 40 μg total RNA was mixed with a 17 mer dT oligo (Sigma) and reverse transcribed in the presence of dNTPs containing 5-(3-aminoallyl)-2'deoxyuridine-5'triophosphate, (aa-dUTP) (Sigma) with SuperScript II^® ^reverse transcriptase (Invitrogen) and conjugated to either Cy3 or Cy5 post-labeling reactive dyes (GE Healthcare Biosciences) using a previously published amino-allyl labeling technique [69-71]. Once re-suspended in hybridization buffer, the labeled samples from the three control and three infected mice were hybridized in pairs to the mouse exonic evidence based oligo (meebo) 36 K array. The MEEBO array is an open source collection of probes designed to yield information on ~25,000 mouse genes. The collection includes probes for ~25,000 constitutive exons, ~4,000 alternately spliced exons, and > 5,000 mRNAs. Further information can be found at the web-links in the references section [72-74]. Specific details of the hybridization procedure can be found using the web-link at reference [75].

Hybridized arrays were scanned with a dual-laser Axon GenePix 4000A scanner (Axon Instruments) adjusting the individual photo-multiplier tube (PMT) settings for each channel according to manufacturers instructions. Spot finding was done with BlueFuse 3.2 [76] which uses a Bayesian approach where a single parametric model represents the microarray data generation process, including sources of noise. The alignment of grids was checked prior to spot finding, but no manual intervention or flagging was performed. The BlueFuse output includes a single measure of intensity for each channel, as well as a spot quality measure (confidence), present call and quality flag.

The data from BlueFuse were analyzed using R Version 2.2.0 for Windows [77], limmaGUI Version 1.4.0 [78] and limma Version 2.3.3 [79] MA plots and M box plots, generated with limmaGUI, showed that normalization was required. Lowess normalization within print tips was used to normalize the data within arrays, but between arrays normalization was not required [80]. Using limma the differences between experimental groups for each gene were determined by specifying and estimating the parameters of a linear model [81]. The significance of the estimates was found from a moderated t-statistic based on a global variance computed from all the genes using an empirical Bayes approach. Several methods are available for adjusting for multiple testing. In this analysis we controlled the FDR (false discovery rate) to be less than 1%. The posterior log-odds, B, that a gene is differentially expressed was calculated and used to rank the genes.

The practice of excluding (filtering) poor quality spots reduces power and efficiency and may lead to bias. Various strategies for excluding or weighting spots using the BlueFuse confidence measure and quality flag were compared between two randomly chosen subsets of data created from the 5858 genes which occur more than once on the MEEBO array. Using the B statistic, the rank correlation between the two subsets was calculated for each strategy. The percentage of spots in the top *N *ranked genes common to both subsets was calculated for all values of *N *and plotted against *N*. Using these two criteria, weighting poor quality spots by the square root or arcsine of the BlueFuse confidence measure produced results that were the most concordant between the two subsets. In the analyses presented here all the spots corresponding to genes were included in the analysis but were weighted by the square root of the BlueFuse confidence measure.

### Histopathology

Intact brains from SPF, SW mice were removed and placed in paraformaldehyde overnight. Tissue was embedded in paraffin; serial coronal sections (10 μm) were stained with hematoxylin and eosin [82]. The presence of tissue cysts, perivascular cuffing, and diffuse lymphocytic infiltrate confirmed toxoplasmic encephalitis in infected mice. Meningitis was scored 0 = none, 1 = mild, 2 = moderate, 3 = severe; Perivascular cuffing was scored 0 = none, 1 = mild, 2 = moderate, 3 = severe; Inflammatory lesions were scored according to the number identified in one section; Calcifications were scored according to the number identified in one section; Cysts were scored according to the number identified in one section; Inflammation with cysts was scored as the number of cysts adjacent to inflammatory lesion – other cysts were in apparently normal brain. There was no necrosis in any of the brains. Interpretation of histopathology was performed by pathologists (FR and RW) without knowledge of the infection status or treatments of mice. Heart and aorta were also evaluated in a subset of these mice.

### Immunohistochemistry and electron microscopy

#### Immunostaining for T cells and activated microglia

Frozen tissue samples from SW mice that were part of the same groups of mice used for behavioral, neurologic and other histopathologic studies were prepared in 10 μm sections were fixed in 4% paraformaldehyde for 10 minutes and washed with 4% PBS. Avidin/Biotin blocking solution (SP-2001, Vector Laboratories) and 3% hydrogen peroxide (H325-500, Fisher Scientific) were used to block endogenous enzyme activity. The tissue sections were incubated with anti-mouse CD4 (1 μg/ml, GK1.5), CD8 (4 um/ml, Cat#553027 from PharMingen), CD11b (2–20 μg/ml MAB1458 from Chemicon International) antibodies and biotinylated ricinus communis agglutinin I (10 ug/ml, B-1085, Vector Laboratories) at 4°C over night. Biotinylated anti-mouse IgG (10 μg/ml, BA4001, Vector Laboratories) was applied and incubated for 30 minutes. Antigen-antibody binding was detected with an ABC kit (PK-6100, Vector Laboratories) and DAB substrate chromogen system (SK-4100, Vector Laboratories). Slides, counterstained with hematoxylin, were evaluated using a light microscope. Brain tissue from 5 control and 5 infected mice were compared.

#### Immunostaining for tachyzoites and bradyzoites and identification of host plasma cells

These studies were performed as previously described [77]. Sections were double labeled with rabbit anti-SAG1 (tachyzoite specific) and mouse anti-BAG1 (bradyzoite specific) and visualized using anti-rabbit-Ig conjugate to fluorescein isothiocyanate and anti-mouse IG conjugated to Texas red. In addition, the anti-mouse Ig also labeled the immunoglobulins within the plasma cells thus identifying their location. Additional sections were stained with anti-SAG1 and antibody CC2 (stains tissue cyst wall).

#### Electron microscopy

The images of these sections were prepared as described previously for the manuscript using non-SPF, SW mice that were chronically infected with *T. gondii *[83]. They are included herein to provide additional insights concerning the pathogenesis of this infection in the context of the immunostaining performed herein [50,51].

### Myelin and other special neuronal staining and histopathology

Sections of the brains of SW mice that were part of the other studies also were examined with hematoxylin and eosin [82], trichrome, Bodian, Bielshowsky and Nissl stains as well as with immunohistochemical stains for neurofilament and amyloid precursor protein. Evidence of neuronal loss, demyelination, axonal damage or widespread microglial activation was sought using light microscopy.

### Lipoprotein and SAA analysis

To determine whether there was evidence of a systemic inflammatory process altering plasma lipoproteins, plasma lipoproteins in sera from the same SW mice that were included in the behavioral and neurologic and histopathologic studies were separated by FPLC on tandem Superose 6 columns and the cholesterol in the fractions analyzed using a kit from Roche Molecular Diagnostics. SAA in the plasma was determined by immunoblotting of using a rabbit anti-mouse SAA antibody [84].

### Knockout mice

To determine whether certain cytokines and innate immune responses were necessary and sufficient for the types of inflammatory responses observed in neuronal tissues of the SW mice that formed the majority of the present studies, mice without these cytokines or innate immune responses, i.e., IL4, IL6, IL13, or *Nramp *knockout mice, in separate studies were infected, maintained and studied as described previously [82]. Histopathology and its analysis was performed exactly as above by one of the same pathologists (FR) who evaluated the histopathology of the SW mice. In these experiments, infection was with the clonal type II Beverly strain of *T. gondii*.

### Chronically infected and uninfected control Balb/c mice

Chronically infected and uninfected control Balb/c mice were maintained in a SPF colony in our (YS) laboratory [85]. The mice were either infected with Me49 *T. gondii *or were uninfected controls and were processed for histopathology as described above and evaluated by pathologists including one of those (RW) who characterized pathology in the SW mice without knowledge of the infection status or treatment of the mice. Brain tissue was obtained at 2 and 5–6 months following initiation of infection.

### Treatment of mice with sulfadiazine

To determine whether tachyzoites contributed to the inflammatory process observed, mice that had been infected for 5 months were treated with sulfadiazine (100 mg/ml) in their drinking water for 4 months. This treatment is known to eliminate tachyzoites from brain but not to eliminate cysts (McLeod, Unpublished data).

### Treatment of mice with hamster polyclonal antibody to PD-L1 or hamster isotype-control

This was administered as described [86] and as had been shown in earlier studies to ablate PD-L1 function in mice.

### Statistical analysis of behavioral and neurologic findings

Statistical analysis was conducted using Stata Version 9 (Stata Corp., College Station, TX). Cognitive/behavioral assessments were summarized as mean ± SD unless otherwise noted. For comparisons between infected/uninfected mice, the Wilcoxon rank-sum test, Kruskal-Wallis test, or Fisher's exact test, was used as appropriate. An overall behavioral measure was created using the total number of behaviors exhibited. For those behaviors that had a continuous distribution (e.g., latency to move), the median value was used as the cutoff for creating a dichotomous variable. Additionally, some ordinal scales were dichotomized (i.e., absent = 0 or present = 1).

### Statistical analysis of histopathologic findings

Statistical analysis was conducted using Stata Version 9 (Stata Corp., College Station, TX). Histopathological data were summarized as mean ± SD unless otherwise noted. For comparisons between infected/uninfected or treated/untreated mice, the Wilcoxon rank-sum test, Kruskal-Wallis test, or Fisher's exact test, was used as appropriate. Spearman rank correlation coefficients (r) were calculated when examining the associations between pathological findings and behavioral measures. An overall measure of disease burden based on the pathological findings was created by taking the sum across types of findings (cuffing, inflammation, calcifications, cysts, and inflammation with cysts) and brain regions (Co, D, H, and Ce). Friedman's test, a nonparametric alternative to the repeated measures analysis of variance (ANOVA), was used for comparisons between brain regions among infected mice only. The pathological findings in the four areas of the brain were considered after summing across types of findings.

## Results

### Appearance, behavior, and neurological findings in the initial cohort

**Figure 1:**
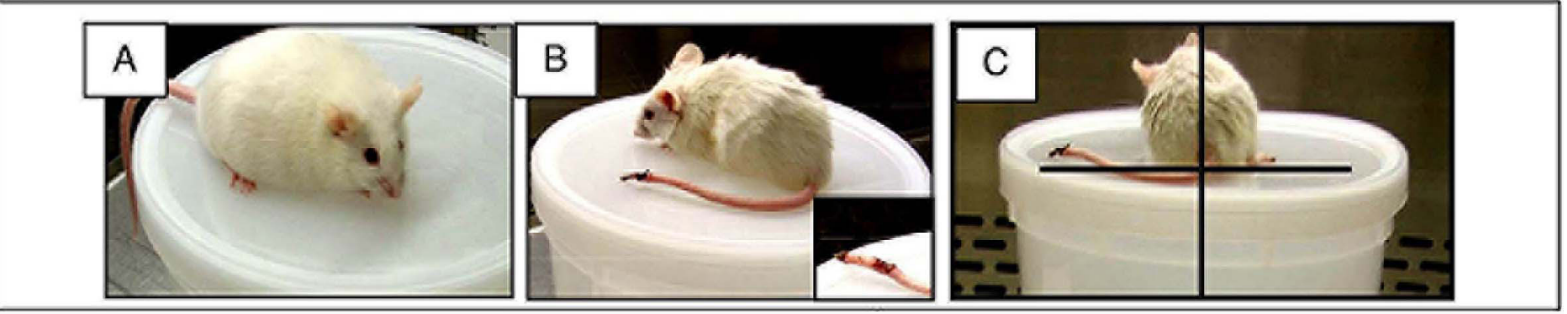
**Appearance of eleven-month-old Specific Pathogen Free (SPF) mice that are uninfected and chronically infected with *T. gondii***. (A) Eleven month old uninfected Swiss Webster mouse. (B) Eleven month old infected female mouse from the same SPF colony ten months after acquisition of *T. gondii *infection. Note hunched body position, poor grooming, piloerection, reduced body mass, and tail wounding. (C) Chronically infected SPF mouse with abnormal body position.

**Figure 2:**
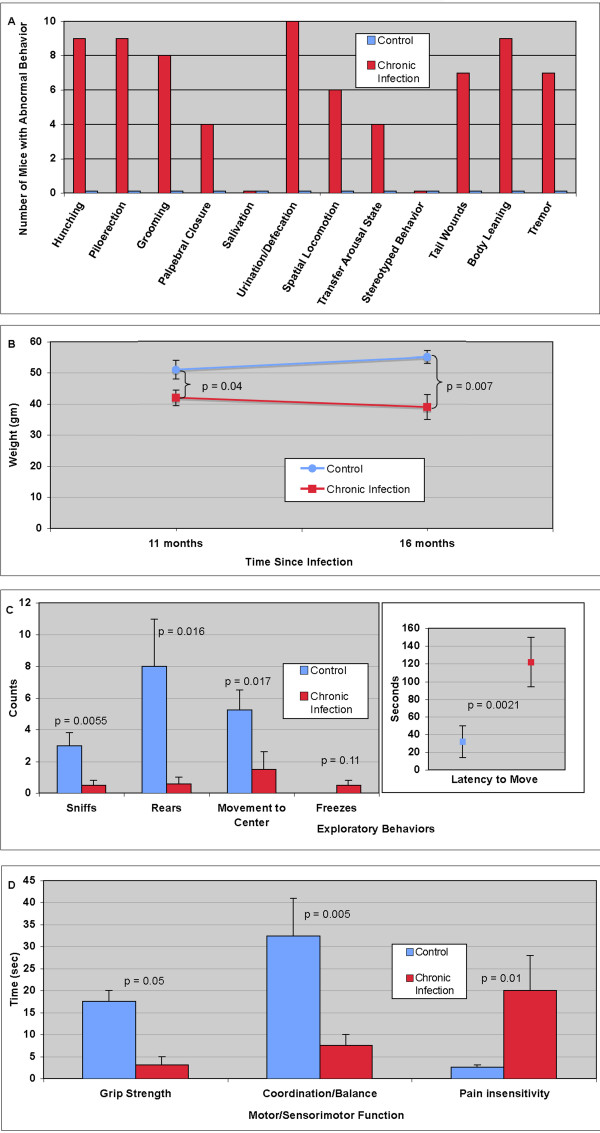
**Behavioral and Neurologic Data of eleven-month-old Specific Pathogen Free (SPF) mice that are uninfected and chronically infected with *T. gondii***. (A) Number of mice with abnormal behavior or neurologic findings. (B) Weight of uninfected and chronically infected mice at eleven (p = 0.04) and sixteen months (p = 0.007) after infection. Error bars indicate one standard error. (C) Exploratory behaviors in mice twelve months after infection. Error bars indicate one standard error. (D) Motor and sensorimotor function in uninfected and infected mice eleven to sixteen months after infection. Error bars indicate one standard error. n = 6 control; n = 10 infected in all cases.

Decrements in behavioral and neurological function in infected mice were first observed in a pilot study and then systematically characterized for all assessments in the "initial cohort" (Table 1; Figures 1, 2; movie – Additional file 1). These results were observed in carefully monitored and characterized SPF mice chronically infected with *T. gondii*. Standard measures of appearance (grooming, body position, piloerection, tail wounding, gait, tremor, and weight), behavior (exploration, freezing, and rearing), and neurologic function (grip strength, motor coordination and balance, and pain sensitivity) were assessed. Abnormalities were noted in the pilot experiment at 5 and 11 months after infection and in the initial cohort at 11 to 16 months after infection.

**Table 1:** Neurologic and behavioral findings in SPF SW female mice infected at 7 months of age and tested at 12 months of age

APPEARANCE									LOCOMOTION		ANS	OPEN FIELD ACTIVITY				
Condition	Grooming	Body Position	Piloerection	Tail Damage	Tremor	Palpebral Closure	Lacrimation	Weight (gm)	Spatial Locomotion	Gait	Urination/Defecation	Sniffs	Rears	Center	Freezing	Latency to move (sec)

Control	Good	-	-	-	-	-	-	39.4	Active	-	-	2	20	4	0	4
Control	Good	-	-	-	-	-	-	52.3	Active	-	-	4	4	2	0	22
Control	Good	-	-	-	-	-	-	45.0	Active	-	-	6	13	3	0	41
Control	Good	-	-	-	-	-	-	50.5	Active	-	-	1	0	7	0	120
Control	Good	-	-	-	-	-	-	50.6	Active	-	-	4	9	7	0	10
Control	Good	-	-	-	-	-	-	58.5	Active	-	-	1	2	9	0	1

Infected	Poor	+	+	+	+	-	-	43.8	Slow	+	+	nd	nd	nd	nd	nd
Infected	Good	+	+	+	-	-	-	41.2	Active	+	+	0	0	0	0	180
Infected	Very Poor	+	-	+	+	-	-	42.0	None	+	+	0	0	0	0	180
Infected	Very Poor	+	+	-	+	+	+	35.3	None	+	+	nd	nd	nd	nd	nd
Infected	Good	-	+	+	-	-	-	34.9	Active	-	+	0	0	0	0	180
Infected	Very Poor	+	+	-	+	+	+	34.8	Slow	+	+	0	0	0	0	180
Infected	Poor	+	+	+	+	+	+	35.6	Slow	+	+	1	3	1	1	24
Infected	Sub-normal	+	+	-	+	+	+	36.2	Active	-	+	0	2	10	1	6
Infected	Very Poor	+	+	+	-	-	+	58.0	None	-	+	0	0	0	0	180
Infected	Poor	+	+	+	+	-	-	54.2	Slow	-	+	2	0	1	2	56

P-values	0.018	0.001	0.001	0.011	0.011	0.23	0.093	0.059	0.035	0.034	< 0.001	0.0055	0.016	0.017	0.11	0.021

+ = abnormal, - = normal, nd = not done. Figure 1 shows representative examples of abnormal grooming, body position, piloerection, and tail damage (Figure 1 B, C) in infected mice when compared with control shown in Figure 1A.

In Figure 1, the normal, sleek appearance of the uninfected control mouse (Figure 1A) contrasts with the ruffled fur and tail wound of the infected mouse in Figure 1B and the tilted posture of the infected mouse in Figure 1C. The number of mice with abnormal findings in various aspects of their appearance are shown in Figure 2A. With the exception of stereotyped behavior, palpebral closure, lacrimation, and salivation, the difference between infected and uninfected mice in all of the categories (grooming, body position, piloerection, tail wounds, and tremor) was statistically significant (Table 1, Figure 2A).

In this initial cohort, chronically infected mice weighed less than uninfected mice at both 11 and 16 months after infection (Figure 2B; p = 0.04, p = 0.007). In addition, the infected mice lost weight over time while the uninfected mice gained weight. Locomotion including transfer arousal and gait were both significantly different in infected mice than in uninfected mice (Table 1; p = 0.035, p = 0.034). A decrease in autonomic nervous system function of infected mice, measured by increased urination and defecation during brief periods of handling, was also statistically significant when compared to uninfected mice (Table 1; p < 0.001). Exploratory movements including sniffs, rears, movement to the center of an open field were significantly less and latency to move significantly greater in infected mice (Figure 2C; p = 0.0055, p = 0.016, p = 0.017, p = 0.021) and only infected mice exhibited freezing behavior, although it did not achieve statistical significance.

Measures of motor and sensorimotor function also were impaired in infected mice (Figure 2D). Infected mice displayed a statistically significant increase in the time it took to remove/attempt to remove a paperclip attached to their tail (indicating decreased pain sensitivity as a sensorimotor measure; p = 0.003) and a statistically significant decrease in the length of time that both grip strength and balance were maintained (p = 0.005; p = 0.01; please also see Movie, Additional file 1 in on-line supplement).

### Kinetics of development of behavioral and neurologic abnormalities in two additional studies (replicates of each other)

To characterize the kinetics of development of these findings and to make certain a subset of our initial observations were reproducible, two additional experiments with 5 infected mice and 5 control mice in each experiment were performed. In these replicate studies, appearance, gait, exploratory behavior, and pain sensitivity (paperclip test) were evaluated. In both of the replicate experiments, abnormalities in appearance, similar to those found in the initial cohort, were found in some of the infected mice as early as 3 months post infection, the initial time observations were made. By 5 months after infection, four of the ten infected mice in the combined replicate studies had died, two had pronounced gait abnormalities involving their lower extremities, and all but one of the remaining mice had more subtle abnormalities in gait. These latter mice moved their lower extremities more slowly and less facilely and had diminished exploratory behavior when placed in a novel, open environment. One of the three mice with the subtler gait abnormalities had a posture where it was tilted to one side. At the time of the initial observations (3 months post infection), infected mice were taking longer, on average, to notice the paper clip attached to their tail, although this did not reach statistical significance (14.9 ± 13.0 vs. 6.4 ± 8.7 seconds in controls, p = 0.39). At five months, the difference was larger and statistically significant (20.5 ± 11.1 vs. 8.4 ± 7.0 seconds, p = 0.039). No tail lesions, which were noted in the initial cohort, were seen in any of these ten mice before the experiment was terminated.

### Brain MRIs of chronically infected mice have mild to moderate ventricular dilatation

**Figure 3:**
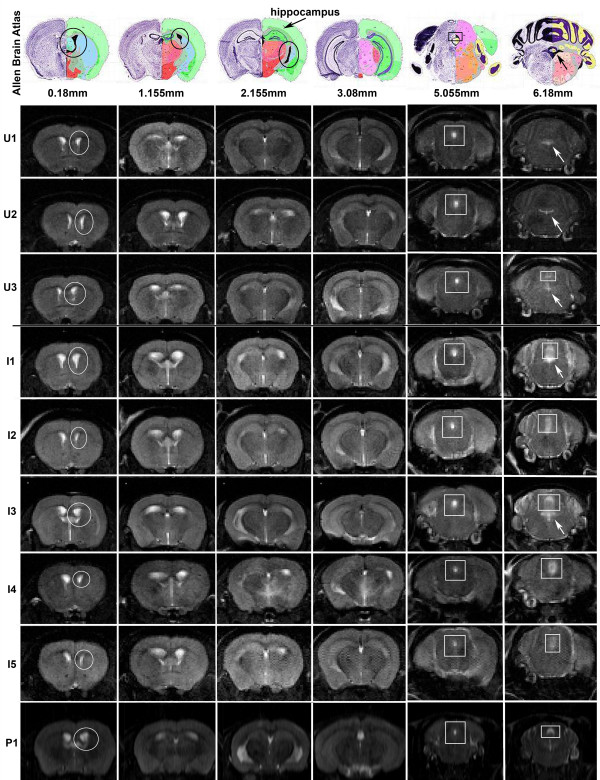
**MRI findings in chronically infected mice and in uninfected mice**. T2 weighted MRIs from uninfected mice (U1-3), chronically infected mice (I1-5), and one infected mouse studied in a pilot experiment at a different time (P1). MRIs were obtained when mice were 1 year old and infected mice had been infected for 8 months. In infected mice there was minimal to moderate ventricular dilation. Images from the Allen Brain Atlas are provided in the top panel for reference to the brain region imaged. Lateral ventricles are marked with a circle and the 3^rd ^ventricle/aqueduct of Sylvius is marked with a square. Unless otherwise noted, the 4^th ^ventricle is marked with an arrow. T2 weighted MRI of the brain in infected and age-matched control mice show an increase of size in the lateral ventricle of infected mice (p = 0.034 approximately 1.155 mm caudal to bregma) and an increase in 3^rd ^ventricle size (marked by a square) of infected mice (far right panel, p = 0.002). In 4 of 6 infected mice there is right-left brain asymmetry (I1, I3, I4, I5). The major difference between the infected and the uninfected MRIs was ventricular dilatation and some asymmetry present in brain parenchyma of infected mice.

To determine whether noninvasive neuroimaging could identify any abnormalities and if so their anatomic distribution, brain MRIs were performed for 6 chronically infected mice (8 months after infection [n = 5] or 12 months after infection in the case of the pilot [n = 1]) and 3 control mice. Representative frames from the MRIs are in Figure 3. T1 weighted images showed no clear abnormalities between the uninfected and infected mice. T2 weighted images (Figure 3) for the control, uninfected mice revealed no abnormalities other than very slight lateral ventricular dilatation. Similar MRI images of the brains of the infected mice, however, showed no (n = 1), mild (n = 1) and moderate (n = 4) lateral ventricular dilatation, which can be seen in the lateral ventricles approximately 0 to 1 mm caudal to bregma when compared to the MRIs from the uninfected mice. Quantitation of the differences in ventricular size at approximately 1 mm caudal to bregma revealed smaller ventricles in the uninfected as compared with the infected mice, which was statistically significant (p = 0.03). In addition, the areas adjacent to the aqueduct of Sylvius had enlargement of the ventricles and periventricular and periaqueductal changes (Figure 3). At approximately 6 mm caudal to bregma, the difference in ventricular size between the uninfected and infected mice also was statistically significant (p = 0.002). No parenchymal abnormalities were noted in two of the infected mice, and asymmetry of uptake of contrast in the cortex in the T2 weighted images was noted in the other four infected mice.

### Correlation of brain weight and neurologic findings

Studies of brain weight and correlation with behavioral and neurologic studies were performed to examine whether chronic *T. gondii *causes loss of brain parenchyma and neuronal cells and whether behavioral and neurologic abnormalities correlated. Such neuronal cell loss was suggested by the brain MRI studies and the increase of GFAP message in the full genome microarrays which reflected neuronal cell injury (see below). In the first behavioral and neurologic experiment, brain weight was not measured. In the second and third replicate experiments described earlier, the mean ± sd [range] of brain weight for infected and control groups were 0.42 ± 0.04 [.385–.466] g and 0.46 ± 0.03 [0.427–0.510] g, respectively (p = 0.065). Further, the infected mice that had more obvious gait abnormalities had the smallest brains. In these experiments a score for abnormal neurologic function and movement pattern was defined as follows: normal gait and exploratory pattern was scored as 0, mild incoordination of gait was scored a 1 or 2, moderate incoordination was scored 3–7 and severe disability in moving one or both hind legs was scored 8–10, with the higher number the most severe. All of the uninfected mice had a score of 0. The uninfected mice quickly moved from the center of the open field to the perimeter usually moving around the perimeter in one direction exploring it. There were no neurologic abnormalities and no association of body size with brain weight present in the 10 uninfected mice (r = -0.024, 95% CI (-0.64, 0.62), p = 0.95). In contrast, all but one of the infected mice moved much less and had a less organized movement pattern around the perimeter of the testing area, with greater severity of that pattern associated with abnormal movement of the hind extremities. The correlation coefficient for the association of abnormal neurologic examination and movement pattern (i.e., higher score as described above) with diminished brain weight in infected mice was -0.53 (95% CI (-0.81, -0.05), p = 0.035).

### Microarrays reflect inflammation and increased expression of CD36 and PD1L, GFAP, ubiquitin ligase, and C1q

To determine molecular mechanisms whereby *T. gondii *alters brain cell functions and reflects the pathogenic process, full genome microarrays were performed using the MEEBO array containing ~36000 probes representing ~25000 genes. All the probes that were significantly differentially expressed are summarized and listed individually in Table 2. There were 326 significant probes (corresponding to 311 different genes) with an adjusted P-value less than 0.01 and a posterior log odds of differential gene expression greater than 2 (i.e. a posterior probability greater than 0.88). All these genes showed greater expression in the chronically infected brains and many are associated with the immune response (Table 2[87,88]). There were no significantly downregulated genes. The microarray results are consistent with an inflammatory process involving immunoglobulin and B cells and interferon gamma production. In addition, there was increased expression of the Suppression of Cytokine Signaling (SOCS), CD36, and PD-1L genes and others including C1q. GFAP expression reflects astrocyte response to neuronal cell injury. Ubiquitin ligase expression is increased, likely reflecting effects on host cell protein processing.

**Table 2:** Genes upregulated in the brains of mice infected with *T. gondii *for a year

**Function**	Gene* [Number/ID]^†^
Antibody	Ig heavy chain V regions [116]
	
	Ig kappa V regions [119]
	
	Ig lambda 1 germline V region (*Igl-V1, 1810027O01Rik, IgL*) [V00811]
	
	Ig alpha chain (*IGHV1S44*) [M19402]
	
	Rearranged IgA-chain gene, V region (*IGHV1S46*) [M20774]
	
	IgG-1 gene, D-J-C region: 3' exon for secreted form (*Igh-4, IgG1*) [J00453]
	
	Ig germline D-J-C region alpha gene and secreted tail (*Igh-VJ558, AI893585, MGC118142, MGC6727, Igh-A (1g2), Vh186.2/Jh2*) [J00475]
	
	Ig gamma2a-b(c57bl/6 allele) c gene and secreted tail (*IGHG2C*) [J00479]
	
	Immunoglobulin joining chain (*Igj, 9530090F24Rik, AI323815, Jch*) [NM_152839]

Antigen presentation	MHC-I Similar to H-2 MHC-I antigen, D-37 alpha chain precursor (*C920025E04Rik*) [AK083387]
	
	MHC-I Transporter 2, ATP-binding cassette, sub-family B (MDR/TAP) (*Tap2, ABC18, AI462429, APT2, Abcb3, Ham-2, Ham2, MTP2, PSF2, RING11, Tap-2, Y1, jas*) [NM_011530]
	
	MHC-II [22]
	
	MHC-III *(D17H6S56E-3, C6orf27, G7c, NG37*) [NM_138582]
	
	Beta-2 microglobulin (*B2m, Ly-m11, beta2-m*) [NM_009735]

Complement	Complement component 4A/sex limited protein (*C4a, Slp*) GeneCards: Primarily expressed in liver and to a lesser extent in immune cells [NM_011413]
	
	Complement component 4B (*C4b, C4, Ss*) [NM_009780]
	
	Complement component 1, r subcomponent (*C1r, AI132558, C1rb*) [NM_023143]
	
	Complement component C1SB (*C1sb*) [NM_173864]
	
	Complement component 1, q subcomponent, alpha polypeptide (*C1qa, AI255395, C1q*) [NM_007572]
	
	Complement component 1, q subcomponent, C chain (*C1qc, AI385742, C1qg, Ciqc*) [NM_007574]

Major inhibitor of classical complement	Serine (or cysteine) peptidase inhibitor, clade G, member 1 (*Serping1, C1INH, C1nh*) [NM_009776]
	
	Serine (or cysteine) peptidase inhibitor, clade A, member 3N (*Serpina3n, Spi2-2, Spi2.2, Spi2/eb.4*) GeneCards: Although its physiological function is unclear, it can inhibit neutrophil cathepsin G and mast cell chymase. [NM_009252]

GTPase	Interferon inducible GTPase 1 (*Iigp1, 2900074L10Rik, AI046432, AW111922, Iigp*) [AK013785]
	
	Interferon inducible GTPase 1 (*AW111922, Iigp1*) [NM_021792]
	
	Interferon inducible GTPase 2 (*Iigp2, RP24-499A8.1, AI481100, GTPI, (GC102455*) [NM_019440]
	
	Interferon gamma inducible protein 47 (*Ifi47, IRG-47, Iigp4, Iipg4, Olfr56*) [NM_008330]
	
	Interferon gamma induced GTPase (*Igtp, RP24-499A8.4, AW558444*) [NM_018738]
	
	GTPase, IMAP family member 4 (*Ian1, Gimap4, AU019574, E430007K16Rik, IMAP4, MGC11734, mIAN1*), transcript variant 1 GeneCards: Exhibits intrisinic GTPase activity. [NM_174990]

Transcription	Signal transducer and activator of transcription (*Stat1, 2010005J02Rik, AA408197*) GeneCards: Mediates signaling by interferons. Results in induction of a cellular antiviral state. [NM_009283 BC057690]
	
	Transcription factor MafB (v-maf musculoaponeurotic fibrosarcoma oncogene homolog B).(*Mafb, Kreisler, Krml, kr*) GeneCards: Plays a pivotal role in regulating lineage-specific hematopoiesis by repressing ETS1-mediated transcription of erythroid-specific genes in myeloid cells (By similarity). [NM_010658]
	
	Down regulator of transcription 1/Differentially regulated in lymphoid organs and differentiation (*Dr1d*) [AF043513]

Suppression of cytokine signaling	Suppressor of cytokine signaling 1 (*Socs1, Cish1, Cish7, JAB, SOCS-1, SSI-1*) GeneCards: SOCS1 is involved in negative regulation of cytokines that signal through the JAK/STAT3 pathway. Appears to be a major regulator of signaling by interleukin 6 (IL6) and leukemia inhibitory factor (LIF). Regulates interferon-gamma mediated sensory neuron survival (By similarity). [NM_009896]

Proteosome/antigen processing	Proteosome (prosome, macropain) subunit, beta type 8 (large multifunctional peptidase 7) (*Psmb8, Lmp-7, Lmp7*) GeneCards: This subunit is involved in antigen processing to generate class I binding peptides. Stimulated by interferon gamma, involved in the degradation of cytoplasmic antigens for MHC class I antigen presentation pathways. [NM_010724]
	
	Proteosome (prosome, macropain) subunit, beta type 9 (large multifunctional peptidase 2) (*Psmb9, Lmp-2, Lmp2*) GeneCards: stimulated by interferon gamma, involved in the degradation of cytoplasmic antigens for MHC class I antigen presentation pathways. [NM_013585]
	
	Proteasome (prosome, macropain) 28 subunit, alpha (*Psme1, AW413925, MGC113815, PA28a*) GeneCards: Implicated in immunoproteasome assembly and required for efficient antigen processing. The PA28 activator complex enhances the generation of class I binding peptides by altering the cleavage pattern of the proteasome. Induction by interferon gamma. [NM_011189]

Antimicrobial	Secretory leukocyte peptidase inhibitor (*Slpi*) Acid-stable proteinase inhibitor with strong affinities for trypsin, chymotrypsin, elastase, and cathepsin G. Secretory leukoprotease inhibitor, involved in antineutrophil elastase protection at inflammatory sites. [NM_011414]
	
	Defensin related cryptdin 17 (*Defcr17, AU014719, Cryp17*) GeneCards: Acid-stable proteinase inhibitor with strong affinities for trypsin, chymotrypsin, elastase, and cathepsin G. Secretory leukoprotease inhibitor, involved in antineutrophil elastase protection at inflammatory sites [S73391]

Pre-B-cell growth	Bone marrow stromal cell antigen 2 (*Bst2, 2310015I10Rik, C87040, DAMP-1*) GeneCards: May be involved in pre-B-cell growth. [NM_198095]

T lymphocyte proliferation	CD274 antigen/Programmed cell death 1 ligand 1 precursor (*Cd274, B7-H1, PD-L1, Pdcd1l1, Pdcd1lg1*) Genecards: Involved in costimulatory signal, essential for T lymphocyte proliferation and production of IL10 and IFNG, in an IL2-dependent and a PDCD1-independent manner. Interaction with PDCD1 inhibits T-cell proliferation and cytokine production. Up-regulated on T and B cells, dendritic cells, keratinocytes and monocytes after LPS and IFNG activation. Up-regulated in B cells activated by surface Ig cross-linking. [NM_021893]

Lymphocyte activation	Lymphocyte-activation gene 3 (*Lag3, CD223, LAG-3, Ly66*) GeneCards: Involved in lymphocyte activation. Binds to HLA class-II antigens. Highly homologous to CD4, expressed exclusively in activated T and NK lymphocytes, major MHC class 2 ligand potentially involved in the regulation of immune response. [NM_008479]

Macrophage growth	Guanylate nucleotide binding protein 2 (*Gbp2*) Uniprot: Interferon-induced Gbp 2 [NM_010260]
	
	Guanylate nucleotide binding protein 4 (*Gbp4, AW228655, Gbp3*) [NM_018734]
	
	Macrophage activation 2/guanylate-binding protein 4 homolog (*Mpa2, AW228052, Gbp4, KIAA4245, Mag-2, Mpa-2, mKIAA4245*) [NM_008620]
	
	Macrophage activation 2 like (*MPA2l, AI595338*) [NM_194336]
	
	Guanylate nucleotide binding protein 5 (*Gbp5*, *5330409J06Rik*) Uniprot: Interferon-induced Gbp5 [NM_153564]
	
	RIKEN cDNA 5830443L24 gene/guanylate binding protein 8**(***5830443L24Rik, mGBP8*) [NM_029509]
	
	cDNA sequence BC057170/guanylate binding protein like (*BC057170, E430029F06*) [NM_172777]

Immune response/binding	Interferon-induced protein with tetratricopeptide repeats 3 (*Ifit3, Ifi49, MGC107331*) [NM_010501] GO:0000004 biological process unknown GO:0005488 binding GO:0005554 molecular function unknown GO:0006955 immune response GO:0008372 cellular component unknown

Astrocyte protein after trauma	Glial fibrillary acidic protein (*Gfap, AI836096*) GeneCards: Class-III intermediate filament, cell-specific marker; during development of CNS distinguishes astrocytes from other glial cells. Almost exclusively expressed in astrocytes; interacts with S100A1. S100A1 home page: S100A1 and S100B can be isolated as a complex from bovine brain; mixture of S100A1 and S100B inhibited the assembly of tubulin into microtubules [NM_010277]

Chemo-attractants	Chemokine (C-C motif) ligand 5 (*Ccl5, MuRantes, RANTES, SISd, Scya5, TCP228*) GeneCards: Chemoattractant for blood monocytes, memory T helper cells and eosinophils. [NM_013653]
	
	Chemokine (C-C motif) ligand 8/monocyte chemoattractant protein-2 precursor (*Ccl8, RP23-446K18.1, 1810063B20Rik, AB023418, HC14, MCP-2, Mcp2, Scya8*) GeneCards: Interferon gamma induced chemotaxis Attracts monocytes, lymphocytes, basophils, eosinophils [NM_021443]

Cell adhesion/interaction	CD36 antigen/fatty acid translocase (*Cd36, FAT, GPIV, Scarb3*) May function as a cell adhesion molecule. GeneCards: Directly mediates cytoadherence of Plasmodium falciparum parasitized erythrocytes. Mediates free radical production in cerebral ischemia. Publication (Khoury, J Exp Med 2003, 197(12) 1657–1666): CD36, a major pattern recognition receptor, mediates microglial and macrophage response to beta-amyloid, and imply that CD36 plays a key role in the proinflammatory events associated with Alzheimer's disease. [NM_007643]
	
	EGF-like module containing, mucin-like, hormone receptor-like sequence 1/cell surface glycoprotein F4/80/lymphocyte antigen 71 (*Emr1, D7A5-7, EGF-TM7, F4/80, Gpf480, Ly71, TM7LN3*) GeneCards: Could be involved in cell-cell interactions. [NM_010130]
	
	Lectin, galactoside-binding, soluble, 3 binding protein/cyclophilin associated. protein (*Lgals3bp, 90K, CyCAP, MAC-2BP, Ppicap*) GeneCards: Promotes integrin-mediated cell adhesion. May stimulate host defense against viruses and tumor cells. [NM_011150]

Leukocyte integrin/actin signaling	FYN binding protein (*Fyb, ADAP, B630013F22Rik*) GeneCards: Acts as an adapter protein of the FYN and SH2-domain-containing leukocyte protein-76 (SLP76) signaling cascades in T cells. Modulates expression of interleukin-2 (IL-2). [NM_011815]

Free radical production	Cytochrome b-245, beta polypeptide (*Cybb, C88302, Cgd, Nox2, gp91<phox>, gp91phox*) GeneCards: Critical component of the membrane-bound oxidase of phagocytes that generates superoxide. [NM_007807]

Apoptosis retardation	B-cell leukemia/lymphoma 2 related protein A1c(*Bcl2a1*, A1-c c) GeneCards: Retards apoptosis induced by IL-3 deprivation. May function in response of hemopoietic cells to external signals and in maintaining endothelial survival during infection (By similarity). [NM_007535]

ADP ribosylation	Poly (ADP-ribose) polymerase family, member 14/collaborator of STAT6 (*Parp14, 1600029O10Rik, BC021340, KIAA1268, MGC29390, mKIAA1268*) [BC021340]

Unknown	[XM_207778]

* Gene names and symbols from Entrez Gene [87] and function information from GeneCards [88]

^†^Either the number of genes in this category or the accession number

Function of all genes significantly upregulated in the brains of mice infected with *T. gondii *for a year (p-value after adjustment for multiple testing < 0.01). No genes were significantly downregulated. Gene names and symbols were retrieved from Entrez Gene [87] using the accession number and function information from GeneCards [88]. Each distinct gene symbol is included in the column headed Gene, except for the functions Antibody and Antibody presentation, where there were too many similar accession numbers. The Number/ID is in brackets contains the accession number where there were < 4 for a function or else a count of the accession numbers for that function.

### Histopathologic abnormalities and immunohistochemistry

**Figure 4:**
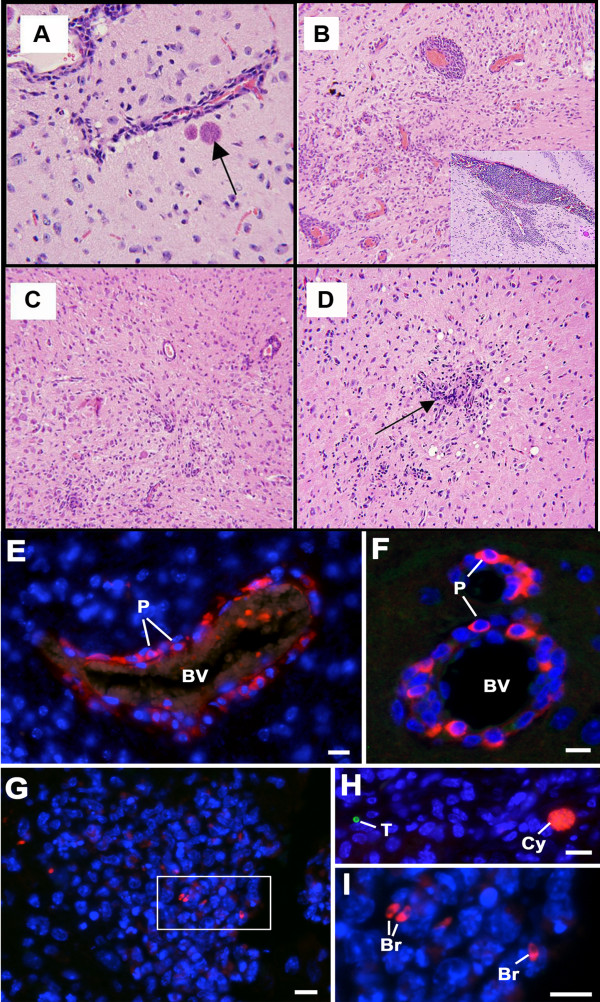
**Inflammation in the brain during chronic *Toxoplasma *infection with only a few bradyzoites and cysts**. (A) Occasional cyst with bradyzoites a short distance from a vessel (arrow), × 250. (B) Medium power view showing perivascular cuffing, × 100. (C) High power view of brain. Note perivascular cuffing and microglial infiltrates. Meningeal infiltrates also occurred (not shown), × 100. (D) Small numbers of microglial nodules (arrow) with diffuse inflammatory infiltrates throughout brain parenchyma, × 100. (E-I) Sections through the brains of chronically infected mice from the behavioral and neurologic studies, double labeled with rabbit anti-SAG1 visualised with anti-rabbit Ig conjugated to fluorescein isothiocyanate (green) and mouse anti-BAG1 visualised with anti-mouse Ig conjugated to Texas red (red). Bars represent 10 μm. (E, F) Examples of blood vessels (BV) cuffed with numerous inflammatory cells in which the plasma cells (P) can be identified by the cross reaction of the anti-mouse Ig with the immunoglobulins within the plasma cell cytoplasm (red), × 400. (G) Low power of a nodule of inflammatory cells in which a few bradyzoites (red) but no tachyzoites (green) could be identified, × 100. (H) A section of brain showing a single tachyzoite (green) and tissue cyst (red) in an area with no inflammatory cells, × 100. (I) Detail from the enclosed area in G showing the cytoplasmic staining of the bradyzoites (Br) with anti-BAG1, × 1000.

To better understand the pathologic processes that caused the abnormal behavior and neurologic function described earlier, histopathology of brain and special immuohistochemistry studies focusing on anatomic brain regions also were performed. Consistent with some of the earlier studies of non-SPF mice [7], histopathological analyses revealed brain abnormalities in the infected SPF mice (Figure 4A–D). These findings included mild to moderate diffuse parenchymal infiltrates of inflammatory cells (predominantly lymphocytes) and with the appearance of microglia (Figure 4A–D). Collections of lymphocytes and plasma cells appeared around blood vessels of different sizes and in the leptomeninges (Figure 4C, D). Focal calcifications were also observed, suggesting a previously healed inflammatory process. Our pathological findings in the context of this chronic infection included rare encysted bradyzoites, the parasite's latent life stage (Figure 4, Table 3). Cysts located within neurons were not associated with an inflammatory response (Figure 4A arrow). Solitary cysts in the brain parenchyma usually were remote from inflammation and calcifications but were occasionally adjacent to, but separate from, perivascular and intra-parenchymal inflammation (Figures 4A arrow).

**Table 3:** Histopathology in brain regions of uninfected and infected mice

Mouse	Meningitis	PV Cuffing				Inflammatory Lesions				Calcifications				Cysts				Inflammation With Cysts			
		
		Co	D	H	Ce	Co	D	H	Ce	Co	D	H	Ce	Co	D	H	Ce	Co	D	H	Ce
U1	0	0	0	0	0	0	0	0	0	0	0	0	0	0	0	0	0	0	0	0	0

U2	0	0	0	0	0	0	0	0	0	0	0	0	0	0	0	0	0	0	0	0	0

U3	0	0	0	0	0	0	0	0	0	0	0	0	0	0	0	0	0	0	0	0	0

U4	0	0	0	0	0	0	0	0	0	0	0	0	0	0	0	0	0	0	0	0	0

U5	0	0	0	0	0	0	0	0	0	0	0	0	0	0	0	0	0	0	0	0	0

U6	0	0	0	0	0	0	0	0	0	0	0	0	0	0	0	0	0	0	0	0	0

I1	2	1	2	2	0	2	4	0	0	4	0	0	4	4	7	1	0	1	0	0	0

I2	1	1	1	2	2	2	0	1	0	0	0	0	4	2	0	0	0	0	0	0	0

I3	1	1	2	1	1	0	1	1	1	0	0	0	1	2	3	2	0	0	0	1	0

I4	2	1	3	2	2	0	1	1	1	0	0	0	0	3	5	1	0	0	1	1	0

I5	3	1	2	1	0	2	2	0	1	0	1	0	3	4	1	2	0	0	0	0	0

I6	3	1	1	3	1	1	1	4	0	0	2	0	3	2	2	2	1	0	0	1	0

I7	3	1	2	2	2	4	3	2	0	0	0	0	2	3	2	2	1	0	0	0	0

I8	1	1	1	2	2	1	0	1	0	0	0	0	1	0	1	1	0	0	0	0	0

**Abbreviations**: U = uninfected, I = infected, U or I is followed by mouse number; Co = Cortex; D = Diencephalon; H = Hippocampus; Ce = Cerebellum

**Scoring System**: Meningitis scored 1 = mild; 2 = moderate; 3 = severe; PV cuffing 1 = mild; 2 = moderate; 3 = severe; Inflammatory Lesions scored according to number of lesions identified in one section; Calcifications Scored according to number of calcifications identified in one section; Cysts scored according to number of cysts identified in one section; Inflammation with cysts scored according to number of cysts adjacent to inflammatory lesion, by implication other cysts were 'sitting' in apparently normal brain.

**Figure 5:**
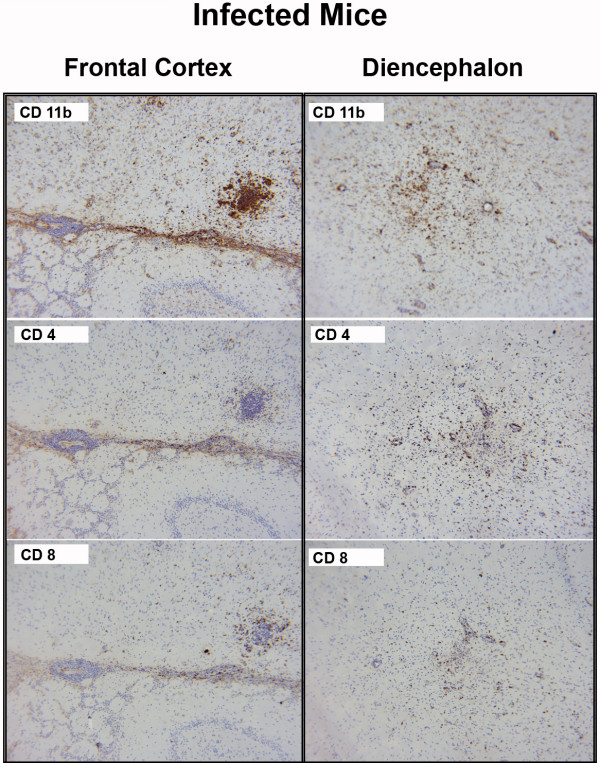
**T cells and microglia in brains of chronically infected mice**. Representative images of one of five infected mice. T lymphocytes were present in nodules and in the perivascular spaces in frontal cortex and diencephalons. CD4 T lymphocytes and microglia and CD8 T lymphocytes ×40. Immunohistochemical stains for CD11 indicate activated microglia. × 100. H & E stains also were performed. In contrast, preparations of brains from 5 uninfected mice revealed no inflammatory cells other than occasional microglial cells (not shown). Secondary antibody control stained tissues had no background staining (not shown).

In the brain, CD4+ T cells, CD8+ T cells, plasmacytoid B cells, and activated microglial cells formed prominent perivascular infiltrates (cuffs around vessels of all sizes) (Figures 4E–F and 5) and diffuse parenchymal infiltrates (Figure 5) which were not present in micrographs of tissues concomitantly prepared from control mice. In chronically infected mice, the individual plasma cells around vessels were identified by the presence of the immunoglobulin within the cytoplasm (Figures 4E, F). In our chronically infected, SPF mice there were rare microglial nodules but no areas of necrosis as seen in immune-compromised persons [89], mice with the C57BL/6J genetic background [36], or various other murine models of immune-deficiencies. In sharp contrast to areas of necrosis in genetically susceptible rodents and immune-compromised persons, manifestations of the infection we observed are associated with substantial chronic inflammatory processes without extracellular organisms.

Sections of brains from chronically infected animals and controls also were studied with trichrome, Bodian, Bielshowsky, Luxol blue, and Nissl stains as well as with immunohistochemical stains for neurofilament and amyloid precursor protein to examine for evidence of neuronal loss, demyelination, axonal damage or widespread microglial activation. However, while the brains of chronically infected mice were smaller than controls, despite the presence of focal areas of chronic inflammation, it was not possible to demonstrate neuronal loss, axonal injury nor extensive demyelination.

As also demonstrated by Ferguson et al in separate studies [50,51], immunostaining only very rarely identified bradyzoites outside cysts (Figure 4G) and only one tachyzoite was identified in all the sections examined from many mice (Figure 4H). However, this confirmed the technique was suitable for the identification of even low numbers of tachyzoites and bradyzoites. (Figures 4E, F). The presence of very few extracellular parasites contrasted with the robust immune response in the brain parenchyma, leptomeninges and around blood vessels.

### Anatomic distribution of lesions and parasite burden demonstrate different areas of predominance of cysts and calcifications

To better understand the basis for the broad range of behavioral and neurologic abnormalities observed, distribution of lesions and parasite burden in various anatomic areas was characterized (Tables 3, 4, 5 and 6). Inflammation and parasite burden were greatest in diencephalon (mean ± SD, median, range: 6.4 ± 3.9, 6, 1–13), then cortex (5.6 ± 3.2, 4.5, 2–12), and then hippocampus (4.9 ± 2.4, 4.5, 3–10). The cerebellum showed less parenchymal inflammation, but had perivascular inflammation and calcifications (4.1 ± 1.1, 4, 3–6). The contrast in magnitude of pathology and cyst number in the specific regions of the brain is shown in Table 3, 5. However, only some of these differences (cyst number and calcifications) reached statistical significance (p < 0.01 and p = 0.02, respectively).

**Table 4:** Comparisons (using Wilcoxon rank-sum test) of infected and uninfected mice

	P-values	Overall p-value*
PV Cuffing (co)	0.0003	0.0012
	
PV Cuffing (d)	0.0010	
	
PV Cuffing (h)	0.0009	
	
PV Cuffing (ce)	0.0090	

Inflammatory lesions (co)	0.0096	0.0012
	
Inflammatory lesions (d)	0.0097	
	
Inflammatory lesions (h)	0.0091	
	
Inflammatory lesions (ce)	0.10	

Calcifications (co)	0.39	0.0037
	
Calcifications (d)	0.20	
	
Calcifications (h)	-	
	
Calcifications (ce)	0.0037	

Cysts (co)	0.0035	0.0012
	
Cysts (d)	0.0037	
	
Cysts (h)	0.0032	
	
Cysts (ce)	0.20	

Inflam w/cysts (co)	0.39	0.051
	
Inflam w/cysts (d)	0.39	
	
Inflam w/cysts (h)	0.10	
	
Inflam w/cysts (ce)	-	

n = 14; *Overall p-value after summing across the 4 brain regions.

**Abbreviations**: co = Cortex; d = Diencephalon; h = Hippocampus; ce = Cerebellum

**Table 5:** Comparisons of the four different brain regions for infected mice only

	Co	D	H	Ce	p-value*
PV Cuffing	1	2	2	1.5	0.12
	1-1	1–3	1–3	0–2	

Inflammation	1.5	1	1	0	0.37
	0–4	0–4	0–4	0–1	

Calcifications	0	0	0	2.5	0.02
	0–4	0–2	0-0	0–4	

Cysts	2.5	2	1.5	0	< 0.01
	0–4	0–7	0–2	0–1	

Inflammation w/cysts	0	0	0	0	0.70
	0–1	0–1	0–1	0-0	

Overall**	4.5	6	4.5	4	0.61
	2–12	1–13	3–10	3–6	

Numbers in table are the median and range; n = 8; there were statistically significant differences in the four regions for calcifications and cysts; * From Friedman's test; **After summing across the 5 findings.

**Abbreviations**: Co = Cortex; D = Diencephalon; H = Hippocampus; Ce = Cerebellum

**Table 6:** Correlations of region of brain involved and behavioral and neurologic findings

	Co	D	H	Ce
Grooming	0.52	0.62	***0.78***	0.54

Body Position	***0.76***	***0.92***	***0.87***	0.66

Piloerection	***0.93***	***0.94***	***0.94***	***0.93***

Tail Damage	***0.84***	***0.71***	***0.74***	***0.89***

Tremor	0.27	0.57	***0.81***	0.39

Palebral Closure	0.17	0.49	0.56	0.14

Lacrimation	0.42	0.57	0.57	0.30

Weight	-0.23	-0.29	-0.28	-0.26

Spatial Locomotion	-0.29	-0.41	-0.69	-0.45

Gait	0.42	0.56	0.38	0.28

Urination	***0.93***	***0.94***	***0.94***	***0.93***

Sniffs	***-0.78***	***-0.71***	-0.53	-0.66

Rears	***-0.71***	-0.56	-0.52	***-0.72***

Center	-0.69	-0.45	-0.41	***-0.71***

Freezing	0.27	0.55	***0.82***	0.42

Latency to Move	0.67	0.44	0.35	0.66

Grip Strength	***-0.78***	-0.57	-0.49	***-0.76***

Pain Insensitivity	0.64	0.65	***0.72***	0.67

Bolded, italicized numbers indicate statistical significance (p < 0.01).

**Abbreviations**: Co = Cortex; D = Diencephalon; H = Hippocampus; Ce = Cerebellum

### Prominent perihippocampal and hippocampal perivascular inflammation

**Figure 6:**
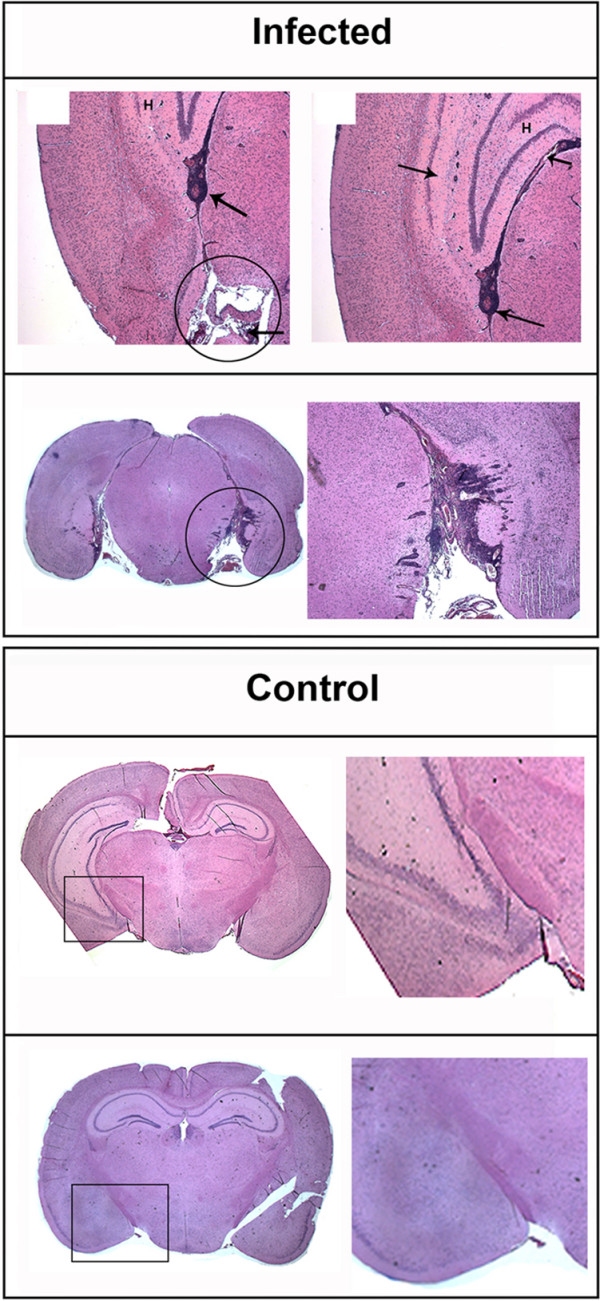
**Perivascular inflammatory infiltrates in vessels that supply the hippocampus (circle) adjacent to the hippocampus (labeled H) and in vessels contiguous to and in the hippocampus (arrows) and at the base of the brain. **No such inflammatory cells are seen in uninfected control mice.

There is an association of hippocampal abnormalities with a number of neurological diseases of humans including Alzheimer's disease, depression, and schizophrenia [56-60]. Thus, it is especially noteworthy that in the chronically infected mice there is an area of pronounced inflammation in the leptomeninges contiguous to the hippocampus, particularly around blood vessels (Figure 6). This was present in all of the eleven chronically infected mice that survived to the end of the studies in the second and the third replicate experiments. It was present in those that had Magnetic Resonance Imaging (MRI), in which tissues were available and this was specifically examined. It was absent in all the thirteen uninfected controls. This process was noted where the posterior cerebral artery bifurcated (Figure 6 circled) and adjacent blood vessels in the leptomeninges traversed the area next to the hippocampus (Figure 6 arrows). The inflammation extended along the blood vessels as they penetrated into the hippocampus (Figure 6, hippocampus marked by arrow). The inflammation was pronounced at the most posterior part of this area but also accompanied vessels continuing anteriorly to the areas above the third ventricle and into the blood supply of the basal ganglia. (Figure 6 right panels provide detail of the pathologic process and show the prominence of this peri-hippocampal process and the absence of the process in the leptomeninges overlying the cortex in this micrograph.) The magnitude of the inflammatory process was comparable to other areas of inflammation in the same mouse, but this region was uniformly involved in each of the eleven mice examined. This more prominent inflammatory process is in the area of the brain associated with short-term memory and spatial orientation.

### Neurologic findings and histopathology correlate

In the initial, first experiments in which very detailed behavioral assessments were made, there also was a significant correlation between the degree of behavioral/neurological abnormalities and magnitude of the parasite burden or inflammatory process (r = 0.87, 95% CI (0.59, 0.96), p = 0.0002; N = 6 infected and 6 uninfected control mice). A correlation of behavioral and neurologic findings with regions of histopathology is listed in Table 6. The presence of calcifications, especially in the cerebellum, without current inflammation suggested that there was an earlier inflammatory process with necrosis that had undergone dystrophic calcification. Lesions along the distribution of the motor pathway and the spinocerebellar tract could also account for gait and tail abnormalities. In the second and third replicate experiments, the infected mice with the most abnormal gait had the most severe brain inflammation and the smallest brains, and a mouse with abnormalities of both legs had substantial inflammatory infiltrate in the corticospinal tract.

### Prolonged treatment with sulfadiazine does not eliminate pathology

A separate group of similar SPF, chronically infected, female SW mice were treated with sulfadiazine or left as untreated, chronically infected, matched controls to better understand whether conversion of bradyzoites egressing from ruptured cysts into tachyzoites might elicit the inflammatory response we observed. Sulfadiazine, which is a competitive analogue of PABA and inhibits tachyzoite growth, but not encysted bradyzoites, does not eliminate all *T. gondii *parasites from congenitally infected or immune-compromised persons [90]. Sulfadiazine treatment administered to our chronically infected mice for 4 months did not significantly modify pole balance (16.4 ± 12.9, range = 0–29 versus 23.6 ± 23.8, range = 2–60; p = 0.68) or grip test results (10.8 ± 5.4, range = 5–19 versus 9.6 ± 11.3, range = 1–29; p = 0.40) or the inflammation and perivascular cuffing in the brain. Treatment with sulfadiazine did reduce, but did not eliminate, the number of cysts (4.8 ± 0.8, range = 4–6 versus 8.0 ± 1.0, range = 7–9; N = 5 mice per group, p < 0.01). These values are for number of cysts in 50 microliters of half a brain, which was homogenized and suspended in 2 ml. Thus, numbers for the whole brain were these numbers of cysts multiplied by 80.

### Heart and large vessels and levels of lipoproteins and inflammatory mediator SAA do not reflect a systemic inflammatory process

To better understand whether the inflammatory process in brain was part of a systemic inflammatory process in chronically infected mice, heart and large vessels, levels of lipoproteins, and the inflammatory mediator SAA were measured. There was no inflammation in the myocardium, and atheromatous plaques were not present in large vessels of mice that had the abnormal neurological findings and brain histopathology (Table 3, 4) in the first experiment (data not shown). Consistent with the absence of this latter finding in our mice, plasma levels of lipoproteins were not altered and assays for the inflammatory mediator SAA from our chronically infected mice showed that there was essentially no SAA in the plasma samples from either control or chronically infected mice (data not shown).

### Effect of antibody to PD-1L on amount of inflammatory infiltrate and number of cysts

Because there was increased expression of PD-1L in whole genome microarrays, and this is a ligand that allows persistent *Lymphochoriomeningitis virus *brain infection by limiting activity of T cells, hamster antibody to PD-1L or isotype-control was administered every 7 days for 21 days. There were three mice tested in each of the following groups: no antibody, isotype-control antibody, and antibody to the PD-1L ligand. Hamster antibody to PD-1L, which has been demonstrated in other studies to abrogate murine PD-1L function, did not significantly decrease the number of cysts (p = 0.12). The median number of cysts were 13, 6, and 5 in the no antibody, isotype-control antibody, and PD-1L antibody groups, respectively. Additionally, inflammation was present in all the groups (p = 0.56). In the mice that received the anti-PD-1L there appeared to be an increased amount of inflammatory infiltrate relative to the numbers of cysts.

### Administration of isolated parasites produces the same pathology

**Figure 7:**
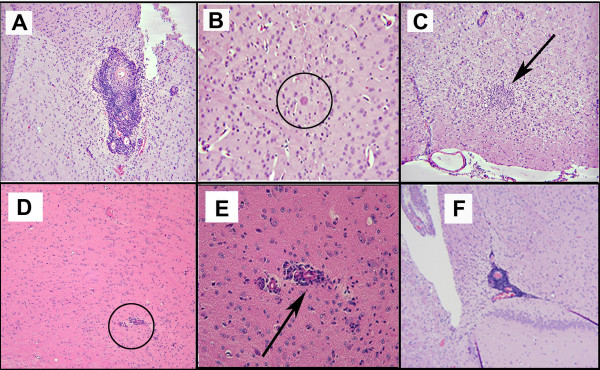
**(A-C) Similar histopathology with perivascular inflammation, isolated cyst ×40, and cluster of microglia in a mouse that is chronically infected, initially infected with parasites without accompanying brain.** A ×40; B ×250; C ×20. (D) Less prominent perivascular cuffing (circle) and small collections of mononuclear cells in Balb/C mouse that is genetically more resistant. E. Increased magnification of area of perivascular inflammation. × 100.(F) Perivascular inflammation × 40 for mouse that had mild lateral ventricular dilatation in MRI.

To address whether infection with concomitant pathogens or parasites administered together with inoculation of brain tissue caused the brain pathology, histopathology of brain from SPF mice chronically infected with Me49 strain (clonal type II) parasites, inoculated without brain tissue, was also studied. The pathology in these mice infected with bradyzoites from isolated cysts (Figure 7A–C) was similar to that described above.

### Genetically resistant BALB/c mice also have perivascular and leptomeningeal inflammation, but there is much less

To determine whether a genetically resistant strain of mouse would have the same type of pathology when chronically infected, chronic infection of BALB/c mice, a strain that is genetically resistant to acute infection and toxoplasmic encephalitis, was also studied. In SPF BALB/c mice infected with clonal type II Me49 parasites for 6 months (N = 5 infected, 3 controls), there were only very rare cysts seen but there was a small amount of perivascular accumulation of inflammatory cells in 4 of the 5 infected mice and leptomeningeal inflammation that was not seen in the controls. These results were similar to studies of brains of BALB/c mice infected for 60 days. Figure 7D is a representative example of this pathology figure 7E is a higher power view of this area of brain. All analyses of tissues of these mice were performed by a pathologist without knowledge of the infection status of the mice from which the tissue was derived.

### Subacute infection in mice with knockout of IL-4, IL-6, IL-13 or NRAMP elicits the same brain histopathology

Because IL-4, IL-6, IL-13 and NRAMP are very important in immunity to toxoplasmosis and could play a pathogenic role in the inflammatory process we observed, mice without IL-4, IL-6, IL-13, or *Nramp*, infected for shorter times, were also studied to determine whether these cytokines or immune processes were necessary and sufficient for the perivascular cuffing and meningeal and parenchymal abnormalities we had observed. These mice also had prominent perivascular cuffing and parenchymal infiltrates, in a distribution similar to that in the outbred mice (FR, CWR, data not shown) indicating that these cytokines and NRAMP were neither necessary nor sufficient to cause the pathology we observed.

### Brain histopathology of mice that had MRIs

To determine whether the type of histopathology we observed would be reflected in a conventional MRI, immediately following the MRIs the brain was removed and fixed in formalin for subsequent histopathologic analysis. The histopathological changes in brain were similar to those described above for the infected mice showing mild to moderate parenchymal inflammation, perivascular cuffing with inflammatory cells, and leptomeningeal inflammation with scattered cysts without inflammation. The uninfected age-matched controls housed in the same colony for 13 months had no brain pathology (p = 0.02 for comparison to infected mice). Figure 4A–D shows the brain histopathology of a representative mouse with only slight ventricular dilatation.

### Electron microscopy and immunostaining of the intact and ruptured tissue cysts

**Figure 8:**
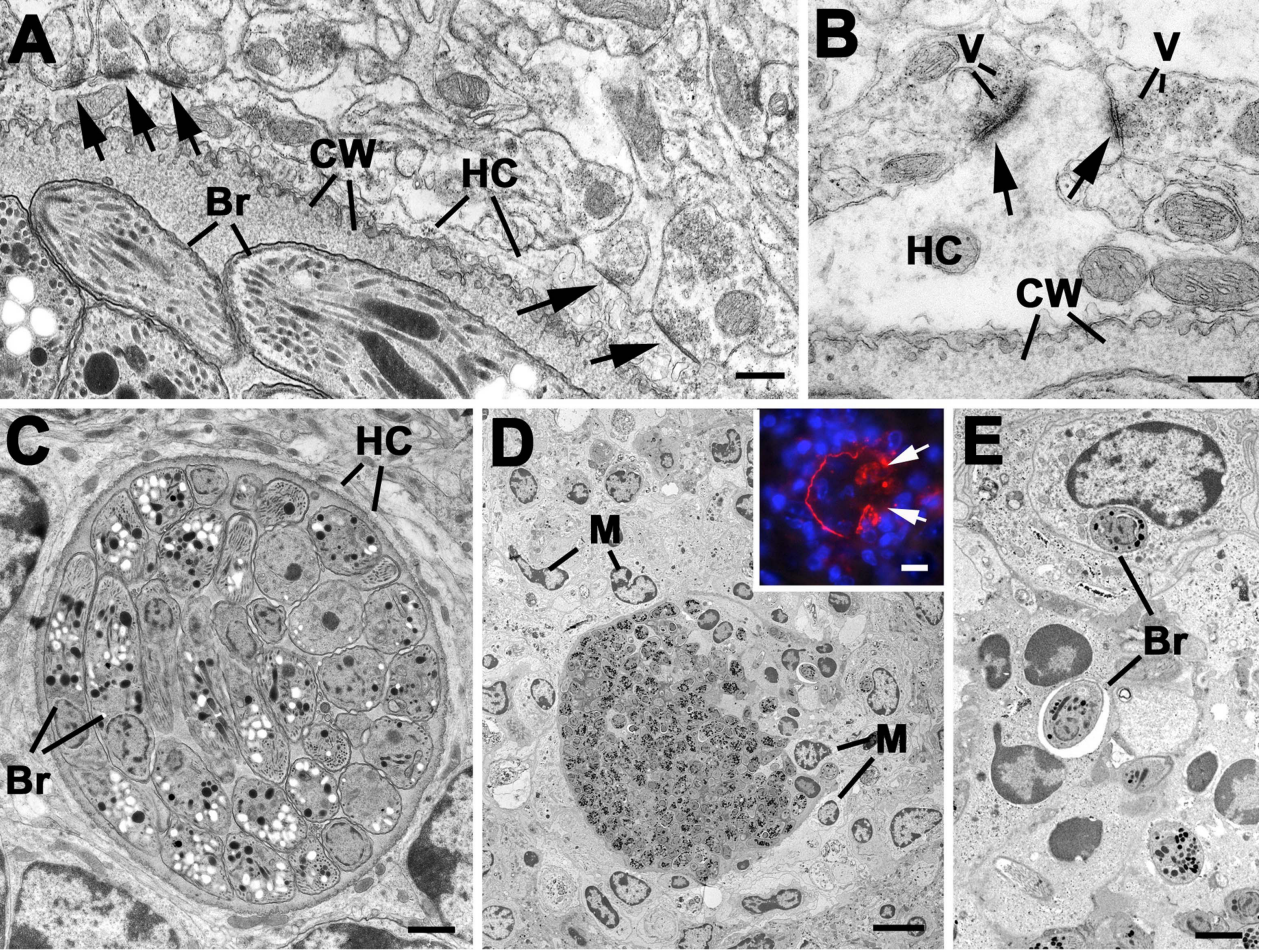
**(A-B) Details of the periphery of tissue cysts from chronically infected mice identifying the host cells (HC) as neurons due to the formation of synapses (arrows).** Based on the structure of the synapse the cysts appear to be within a neuronal dendrite. V – neurosecretory vescicles; CW – cyst wall; Br – bradyzoite. Bars represent 100 nm. (C) Low power of a cyst containing numerous bradyzoites (Br) within a host cell (HC). Note the absence of inflammatory cells. Bar is 1 μm. (D) Low power of a ruptured tissue cyst showing numerous inflammatory cells (M) around and invading through breaks in the cyst wall into the tissue cyst. Bar is 10 μm. Inset: Similar area to that in D immunostained with an antibody to the cyst wall (CC2) showing the disruption to the tissue cyst wall (arrows) and the surrounding inflammatory cells. Bar is 10 μm. (E) Detail from D showing bradyzoites (Br) that had been phagocytised by inflammatory cells. Bar is 1 μm.

The intact cysts were located in neurons identified by the presence of synapses between the host neuron and adjacent neurons (Figure 8A–C). On extremely rare occasions it was possible to observe tissue cyst rupture, which was associated with loss of the host cell (Figure 8D, inset). It was found that mononuclear cells were surrounding the cyst and invading through the ruptured cyst wall. The monocytes were attacking and engulfing the bradyzoites (Figure 8D). The bradyzoites were destroyed before they could convert to tachyzoites.

## Discussion

### Neurologic and behavioral abnormalities in chronically infected mice that acquired primary infection in early adulthood are progressive and persistent

Herein we found that chronic *T. gondii *infection of SPF SW mice following infection in early adulthood alters their appearance, behavior and neurological function, including autonomic function, pain sensation, motor function, balance, spatial, grip strength, coordination, and lack of pain sensation. Many of these findings have not been reported in rodent models before. The abnormalities are clearly more prominent in the SPF SW mice than only alterations in fear, i.e., lack of fear when smelling cat urine ascribed to the amygdala in Balb/c mice by Vyas et al [15] and substantially more global than the subtle, poorer grooming, and decreased exploratory behavior described earlier by others such as Hutchinson [7] and Hay et al [8]. Hutchinson [7] and Hay [8] also described increased movement by chronically infected mice which was not observed in this study. In contrast to only a few specific abnormalities, the global sensorimotor loss is present in the months following infection, is progressive, and persists a year or more after infection. Others with colonies of chronically infected mice, some conventionally housed and some SPF, also observed, but did not report, similar findings (e.g., Louis M. Weiss, M.D., M.Ph., Albert Einstein College of Medicine, Bronx, NY, Jack S. Remington M.D., Stanford University and Palo Alto Medical Research Institute, Palo Alto, California, personal communications to RM, 2006).

### Conventional neuroimaging (MRIs) of chronically infected mice provides insight into identification of consequences of chronic T. gondii infection

Brain MRIs are an initial step in evaluating neurologic and behavioral abnormalities in patients that are similar to those we observed in our chronically infected mice. Therefore, findings in brain MRIs of chronically infected mice were of interest. Brain MRIs were performed with DBA mice chronically infected for 8 months and were not performed with the SW mice with the detailed analysis of behavior and neurologic abnormalities because of logistical constraints. Neurologic abnormalities similar to those in the SW mice also were observed in the chronically infected DBA mice that were imaged (L. Weiss, personal communication, 2006) although they were not quantitated systematically as was done for the SW mice. Histopathology of both the SW and DBA mice (Figures 4, 6, and 7) were subsequently compared and also found to be similar.

With the marked neurologic and behavioral findings that we and others (on further analysis) had noted in chronically infected mice, we expected to find a pathology in brain MRIs during chronic infection that would be reflected in enhancing lesions or areas of necrosis or other discrete pathology in the brain MRIs. Interestingly, and surprisingly, standard non-invasive, objective, imaging measures of brain parenchyma such as standard brain MRIs of mice infected with *T. gondii *for 8 months, with similar histopathology to those mice that had extensive behavioral and neurologic testing, demonstrated only and primarily ventricular enlargement, particularly along the aqueduct of Sylvius, and asymmetry in contrast uptake in T2 weighted scans of infected mice. It seemed remarkable to have profound, repeated, and consistent behavioral/neurological findings without an obvious inflammatory, necrotic or other pathologic changes reflected in the MRI.

The areas in which abnormalities were most prominent in the periaqueductal and periventricular areas are areas that have been noted to be abnormal in some congenitally infected persons, but have not been noted to be preferentially affected in adult acute acquired infections. Interestingly, in congenitally infected infants and in murine models, periaqueductal inflammation leads to hydrocephalous.

**Figure 9:**
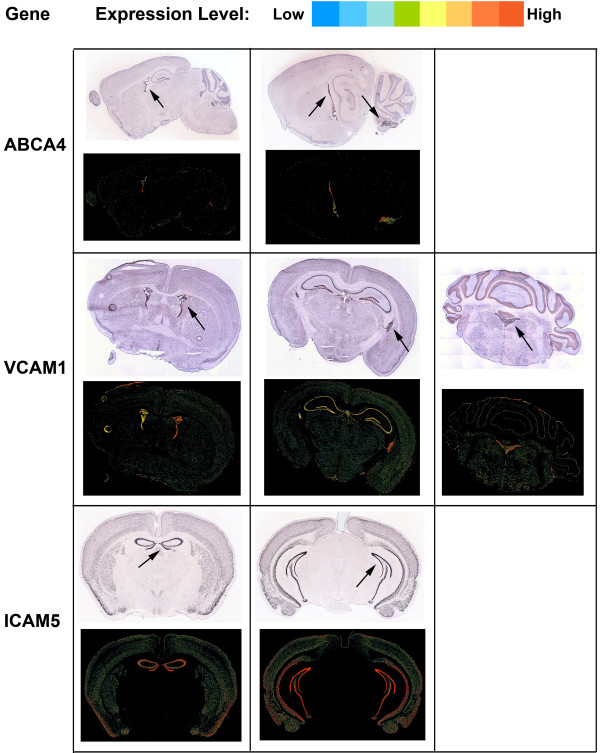
**ABCA4, VCAM and ICAM are expressed in the perihippocampal, hippocampal and periventricular areas.** Images obtained from the Allen Brain Atlas.

It is of interest that expression of the *ABCA4 *gene is localized to the periventricular/perihippocampal area (Figure 9) and that this same area is noted to be prominently involved in the MRIs (Figure 3) and histopathology (Figure 6) of the chronically infected mice. We (RM) recently noted that *ABCA4*, which transports toxic oxidized lipids out of cells, has a susceptibility allele associated with development of hydrocephalus in human congenital toxoplasmosis [39]. This association has a parent of origin inheritance pattern and thus the allele of this gene which is expressed is imprinted *in utero *(i.e., epigenetically modified). Other genes important in pathogenesis of *T. gondii *infection also are expressed in this area of the brain (Figure 9) including VCAM and ICAM. *T. gondii *microneme protein 2 (MIC2) binds to VCAM and is important in parasite attachment during invasion of cells. These findings taken together, suggest that *T. gondii *may secrete molecules with the capacity to epigenetically modify expression of genes important in neurodevelopment or death of infected neurons, or cause bystander cell death, or modify immune responses especially in the area of the brain contiguous to the aqueduct, ventricles and hippocampus [91-93].

Recently developed imaging techniques might, in the future, provide additional, useful insights; e.g. vasculitic changes that may go undetected in standard MRIs may be detected with FLAIR images. Use of functional MRIs and imaging of stem cells with differences in lipids to identify functions of specific regions in human brains [94] and volumetric MRIs to assess hippocampal size and size of other brain regions might be useful and informative in future studies. Ventricular dilatation we observed in MRIs of chronically infected mice, compared with uninfected mice, described herein suggests that there is neuronal cell loss or damage. In similar standard MRIs in persons with neurodegenerative disease affecting the hippocampus, loss of brain parenchyma in the medial aspects of the temporal lobes is manifested by similar increases in lateral ventricular volume.

### Neurologic and behavioral abnormalities correlate with lower brain weight and inflammation, especially in perihippocampal, hippocampal, and periventricular areas, in histopathologic studies of brain of SW mice

To better understand pathology and pathogenesis of the behavioral and neurologic findings we observed, brain weights were correlated with magnitude of neurologic abnormalities. We found that magnitude of the abnormal neurologic findings correlated with lower brain weight. There were no abnormal neurologic findings in uninfected controls. This loss of brain parenchyma among infected mice is consistent with the dilated ventricles seen in the MRIs.

The histopathologic findings observed included individual cysts with no surrounding inflammation remote from perivascular collections of plasma cells, CD4+ and CD8+ T cells and microglial cells, and parenchymal and leptomenigeal collections of T cells and microglia. Peri-hippocampal, hippocampal perivascular and leptomeningeal inflammation were especially prominent (Figure 6). The locations in brain of these collections of cells correlated with and provide an understanding of the anatomic substrate of the specific and broad array of behavioral and neurologic findings (Figure 1 and 2, on-line movie (Additional file 1), Table 1) noted. Mice with the most inflammation had the smallest brains and most abnormal neurologic and behavioral findings. Mice were infected with 100 cysts, a relatively large dose. Since parasite burden seemed to correlate with the severity of disease, it is possible that a lower inocula might better simulate the subclinical outcome representative of natural infections in those who are genetically resistant.

Histopathology provided additional initial insights into pathogenesis of the neurologic findings. Microglial cells, a prominent part of the histopathology we and others [8,50,95] observed in chronically infected mice, also are a feature of neurodegenerative diseases, including Alzheimer's disease, multiple sclerosis, Parkinson's disease, AIDS dementia, and stroke. In these diseases and models for them, microglial cells have been reported to produce neurotoxic molecules, e.g., nitrous oxide, proinflammatory cytokines, particularly TNFα and IL-1β, and reactive oxygen species [96,97] which can cause neuronal cell loss. Recent studies have indicated that mitogen-activated protein kinases mediate glia-induced neuron death [98]. *T. gondii *dense granule protein 10 (GRA10) has been noted to modulate host TAF1 and p38 MAPkinase [99]. The inflammatory infiltrates observed in our murine model of chronic *T. gondii- *induced encephalitis suggest that microglia may affect both infected host neurons and by-stander neurons. Van Eldick et al. have demonstrated that inflammatory mediators also modify clearance of misfolded proteins [98]. Recently, such inflammation has been found to augment prion diseases [55,100].

Similarly, persistent perivascular inflammation, especially involving glial cells [101], is common to a number of neurodegenerative diseases including Multiple Sclerosis, Alzheimer's Disease, Rasmussen's syndrome, Binswanger's disease and subacute sclerosing pancephalitis. In a recent study in mice, perivascular cuffing in the hippocampus increased the normal distance between neural progenitor cells and the vasculature in mice; this increased distance was associated with accelerated rates of cognitive decline in mice [101]. The pronounced perivascular inflammatory process contiguous to the hippocampus seen in all 11 mice from the 3 different experiments where this was specifically examined, suggests that this area of the brain in mice has pronounced pathology. It is of interest that it is the hippocampus which is involved early in Alzheimer's disease, is of smaller volume in schizophrenic patients and those with depression [57-60,102] and is an area of the brain in which its activity in fMRIs is associated with optimism [61].

Special stains did not add insight into pathogenesis with positive findings but rather indicated that in this murine model of chronic toxoplasmosis that there is not demonstratable, extensive focal demyelination, axonal loss or amyloid precursor protein contributing to the pathology.

### Immunostaining for tachyzoites and bradyzoites and review of electronmicrographs provides insights into initiation of the inflammatory process

With immunostaining for tachyzoites and bradyzoites, we noted an absence of tachyzoites and only rare free bradyzoites present with inflammatory lesions in the brains of the same mice studied for behavioral, neurologic, conventional histopathology and microarrays where inflammatory processes were noted. These immunostaining results are replicated in other work described previously [50,51]. In the histopathologic sections, it is noteworthy that the individual cysts were usually remote from the inflammatory process.

Electron micrographs made with brains from conventionally housed, chronically infected, outbred mice have been reported earlier [50,51,103,104] and new images included herein as Figure 8 provide additional insights concerning pathogenesis when placed in the context of the other findings presented herein. In these electron micrographs, *T. gondii *cysts occupied much of the cytoplasm of neuronal cells and the neurons formed synapses (Figure 8A, B), indicating that they are capable of some differentiated functions even with large cysts inside them, although the functioning of such synapses has not been studied. No inflammatory process is noted around the perimeter of the individual cyst when the neuronal membrane was intact.

An earlier study of *T. gondii *in the brains of chronically infected mice, noted that only 2 of ~750 cysts were outside intact host cells [50]. In the rare circumstances when neuronal cell membranes were not present around intact cysts or cysts that had ruptured, there was a marked invasion of inflammatory cells, predominately mononuclear cells but also neutrophils (Figure 8C–E). In our outbred SPF mice chronically infected with *T. gondii *described under histopathology and transcriptome (please see 4.3 and 4.4), immunostaining of brain tissue demonstrated that CD4+ and CD8+ T cells, B cells, and microglia (CD11+) were present in similar areas of parenchymal inflammation and perivascular cuffing (Figure 5). This inflammation occurred whereas brains from uninfected control mice had no inflammatory process. Staining for mouse immunoglobulin shows that antibody-secreting cells are present in the perivascular cellular infiltrates and this and their morphology indicate that these are plasma cells (Figure 4E) and confirmed by electron microscopy [51]. Although the cysts in brains of chronically infected SPF mice have no surrounding inflammation (Figure 1; Table 3) the electronmicrographs in which bradyzoites are associated with inflammatory cells and clearly elicit an immune response, suggest that they may be the life-cycle stage of the organism that causes this immune response once they are outside neurons. This occurs without the need to have bradyzoites convert to the tachyzoite life-cycle stage, which has been demonstrated to cause pathology in immune compromised mice and humans [38,82]. The occasional large cysts that contained parasites within neurons elicit no inflammatory response, although others and we have found that *Toxoplasma *antigens are present in cyst walls (Figure 8D, inset). Whether the neurons containing these large cysts function entirely normally is unknown but they do form synapses [51,103-105].

These collective findings support the following hypothesis: Bradyzoites from cysts in neuronal cells in which host cell membrane is intact elicit no inflammatory response. When the neuronal membranes are disrupted, bradyzoites are ingested by mononuclear cells and these bradyzoites elicit the initial inflammatory response. Then, as described by Van Eldick et al [98], neuronal damage may be associated with cytokines or chemokines from such cells that are known to elicit neurologic damage and loss as a bystander effect in other diseases.

### Transcriptomes of brain from chronically infected SW mice compared with uninfected mice provide additional insights into the molecular pathogenesis of behavioral and neurologic abnormalities and histopathology

Herein, we note that a variety of inflammatory responses and neuronal cell death are reflected in transcriptomes of brains (Table 2) from the chronically infected mice that had behavioral and neurologic testing and histopathologic study. Microarray analysis of gene expression of chronically infected brain tissue and uninfected control brain tissue is consonant with the histological observation that lymphocytes, particularly B cells and antibody secreting plasma cells, are present in the perivascular cuffing. The microarray results showed 326 probes (corresponding to 311 different genes) with a posterior log odds of differential gene expression greater than 2 (i.e. a posterior probability greater than 0.88) and an adjusted P-value less than 0.01. Surprisingly, all these genes showed greater expression in the chronically infected brains and many are associated with the immune response (Table 2). This suggests that the majority of differential expression is probably due to the presence or greater abundance of particular cell types rather than a change in regulation of the cells normally resident in the area.

Expression of chemoattractants *Ccl5 *and *Ccl8*, which promote monocyte and lymphocyte recruitment [106,107], is consistent with the presence of B cells and the dominance of immunoglobulin genes and the perivascular and brain parenchymal inflammatory process. Heavy and light (both kappa and lambda) chain genes comprise two thirds of the list (since cross-hybridization may occur on the array, classification of the variable region family/type is not possible). Consistent with this is the support of B cell growth and lymphocyte activation by *Bst2 *and *Lag3 *respectively [106,108]. The increase in PD-L1 (CD274) ligand, associated with "exhausted" T-cells in a chronic viral infection [86,109], may be a contributing factor that allows latency in chronic *Toxoplasma *infection, although other molecules may also play a role [110]. PD1L has recently been found to tolerize T cells in the brain in acute toxoplasmosis (I. Kahn unpublished data, personal communication to R. McLeod, July 2008) which makes the observation of its increased expression in the brain in chronic murine toxoplasmosis noted herein especially interesting and important. Increased expression of PD-1L suggests that the inflammatory cells that are attracted into the brain may not always be fully functional effector T cells, as demonstrated in LCMV infection [86,109] when this is associated with diminished activity of T cells.

Greater expression of MHC class I, class II and proteosome related genes indicates an increase in antigen processing and presentation. IFN inducible GTPases of both the p47 and p65 classes are consistent with anti-microbial and, perhaps even more specifically, anti-intracellular pathogen activity. IGTP has been shown to be important in limiting *T. gondii *growth in astrocytes [111]. This induction is dependent upon STAT1 for expression [112], a transcription factor that is increased in our study. Counterbalancing the IFN/inflammation is the increase in the cyclophilin associated protein scavenger receptor *Lgals3b *[113]. The increase in CD36 may be relevant to the pathogenesis of behavioral abnormalities and pathology given its role in other conditions including cerebral malaria and Alzheimer's disease [114,115]. CD 36 interacts with TOLL receptors 2/4 with glycoplipid ligands eliciting innate immune responses and influences susceptibility to cerebral malaria and dendritic cell functions [116]. The increase in *Gfap *is consistent with astrocyte response to neuronal cell damage [117].

In addition to the above genes mentioned, the mRNA for subunits of C1q is found in greater abundance compared to the controls, raising the possibility of neuronal loss directly through complement expression and tagging. Previous studies have shown that complement component C1q is upregulated in a range of neurodegenerative diseases including Alzheimer's disease (see for example Fonseca et al [118]). Recent evidence in a mouse model suggests that in the normal development of neural circuits, C1q "tags" inappropriate synaptic connections for selective elimination [119]. Signaling from immature astrocytes appears to induce C1q expression in microglia and neurons and it was hypothesized that reactive astrocytes seen in brain injury may provide a similar signal, ultimately leading to synapse loss and neuronal death. The regulation of complement appears to be balanced as the *slp *increase suggests the need for immune complex clearance concomitant with a *Serping1 *increase, inhibiting complement activation via the classical pathway.

Interestingly, neuronal signaling and brain development pathways recently identified as disrupted with rare structural variants in a genetic analysis of persons with schizophrenia included signal transduction, neuronal activities, nitric oxide signaling, synaptic long-term potentiation, glutamate receptor, ERK/MAPK, phosphatase, axonal guidance, G protein receptor, signaling integrin, and Sonic hedgehog [120]. These mechanistic molecular processes certainly have the potential to be modulated by *T. gondii *or inflammation it elicits as identified in our full genome arrays.

Collectively, these data present a developing picture of the behavioral/neurodegenerative effects of chronic *T. gondii *infection. Clearly this is a dynamic situation and a time series study, combined with the determination of which cells are contributing to the expression increases for a given gene, will be of considerable interest.

### Additional studies of pathogenesis

*T. gondii*, and not adventitious pathogens, or concomitant administration of brain tissue, caused the meningoencephalitis since it was present in SPF mice administered parasites alone. The observation that this meningoencephalitis could be only partially abrogated with sulfadiazine treatment suggests that cyst rupture exposing bradyzoites sequestered from sulfadiazine in cysts, rather than bradyzoite to tachyzoite transformation and tachyzoite growth, might be a primary stimulus for inflammatory processes.

Small collections of perivascular and meningeal inflammatory cells also were observed even in chronically infected, genetically resistant, inbred SPF mice [85]. Interestingly, in a recent report, genetically resistant BALB/c mice infected a month, a time cysts were predominant and tachyzoites were not present, also had a more specific and anatomically localized behavioral abnormality [15].

The presence of the same pathology in mice without NRAMP, IL-4, IL-6, or IL-13, each important in immunity to toxoplasmosis [40], indicates that none of these genes or cytokines are necessary or sufficient to cause this histopathology.

Absence of inflammation outside the central nervous system suggests that behavioral abnormalities and neural lesions probably are not caused by a concomitant, peripheral, inflammatory process. Gazzinelli et al. reported that adult apo E deficient mice previously infected with *T. gondii *in young adulthood (puberty) developed much more (and more severe) atheromatous disease [121]. Our model differs because our mice are apo E sufficient and therefore develop much less atheromatous disease.

### What are the insights from and limitations of murine models in providing a basis for generating hypotheses about how chronic T. gondii infection might affect the brain of humans? What is known about T. gondii infection of the brain in immune competent persons?

Although there are no data from humans in the work described herein, these data from mice raise questions as to whether inflammation in brain caused by *T. gondii *infection could act as a co-factor in the development of neurodegenerative diseases in some genetically susceptible individuals. These data provide insights into regions of the brain most affected which parallel findings in humans [54]. *T. gondii *cysts are seen occasionally in brain from immunologically normal persons at autopsy (FR, unpublished), without any contiguous inflammation, which is much like observations of mice in this study, especially those mouse strains that are genetically more resistant (Figure 7D). The findings in mice of chronic inflammation without visible free tachyzoites and very rare extracellular bradyzoites, which share similarities to responses in neurodegenerative diseases in humans, raise questions as to whether inflammation associated with chronic, cryptic *T. gondii *infection might cause low grade encephalitis or function as a co-factor in common neurodegenerative or other neurologic diseases in some genetically susceptible individuals and extracellular parasites might not be visible. Genetics has a profound influence on susceptibility to acute, subacute and congenital manifestations in this infection both in experimental animals and in humans [33,34,38,39,122,123].

Central nervous system toxoplasmosis in apparently immunologically normal persons has been reported only rarely [124,125]. Toxoplasmic meningo-encephalitis and chronic *T. gondii *antibody production in cerebrospinal fluid of persons with chronic neurologic diseases without known immune-compromise have been reported [124,125], but these findings are not thought to occur commonly. In toxoplasmic encephalitis, which is more frequently but not exclusively reported in immune-compromised individuals, findings are often focal and reflect large areas of necrosis [124] due to unconstrained bradyzoite to tachyzoite switch and then the replication of tachyzoites not contained by a competent immune response leading to encephalitis or necrosis. An inflammatory process resulting in extensive necrosis and proliferation of tachyzoites has been described in some mouse models of infection, e.g., in C57BL/6J mice, but not in immunologically normal SPF outbred mice. Although inflammation around blood vessels and in brain parenchyma has been described in outbred mice infected with some but not all isolates of *T*. gondii earlier [37], this has not previously been well-characterized as to precise location in the brain, extent of inflammation, whether there are directly linked consequences on behavior, or the cause. To our knowledge, many of these findings we noted have not been recognized in other animal models. They certainly are consistent with findings associated with other chronic encephalitidies and neurodegenerative diseases but for the most part they have not been ascribed to chronic toxoplasmosis. There are many cases of encephalitis of unknown etiology [126] but whether and if so how often *T. gondii *might cause chronic encephalitis in humans or non-human animals during chronic infection is not known.

The prominent peri-hippocampal and hippocampal perivascular leptomineageal process in the mice we studied also is of interest because of the association of hippocampal abnormalities in neurodegenerative diseases, depression, and schizophrenia [60]. In schizophrenia there are epigenetic modifications of human DNA [49] that are reminiscent of the manner in which *T. gondii *modifies HERVS in a human neuronal cell line [48]. In Aicardi-Goutieres's Syndrome, there are calcifications in these same areas of brain, e.g., basal ganglia and periventricular areas, that mimic this congenital infection [127]. The recent finding that Aicardi-Goutieres's Syndrome is caused by mutations in a DNAase, *TREX1*, which leads to increased interferon-α production [128], suggests a possible similarity in pathogenesis of Aicardi-Goutieres's syndrome abnormalities and some of those in human congenital toxoplasmosis. Also the occurrence of calcification in congenital toxoplasmosis in basal ganglia and localization of *T. gondii *recrudescence in dopaminergic areas, e.g., the basal ganglia, in immunocompromised persons suggests that there may be a predilection for the parasite to infect these areas in humans [1,90,124]. The hippocampus contains dopaminergic neurons. The prominent involvement of the area around the aqueduct of Sylvius remains unexplained and is of interest in this context. Further, the increased expression of PD-1 ligand and CD36 raise the provocative questions about whether *T. gondii *shares mechanisms to down-modulate critical protective immune functions that also allow latency in infections with certain viruses or cause pro-inflammation contributing to neurodegeneration. The increased expression of C1q raises questions about neuronal synapse remodeling or bystander cell death. The full genome microarrays in the context of our other studies provides insight into gross, cellular, subcellular and molecular pathogenesis and consequences of CNS toxoplasmosis in mice. In so doing, this work provides a foundation for hypotheses for studies to determine whether, and if so where and how *T. gondii *might cause similar changes in the brain of some humans.

**Figure 10:**
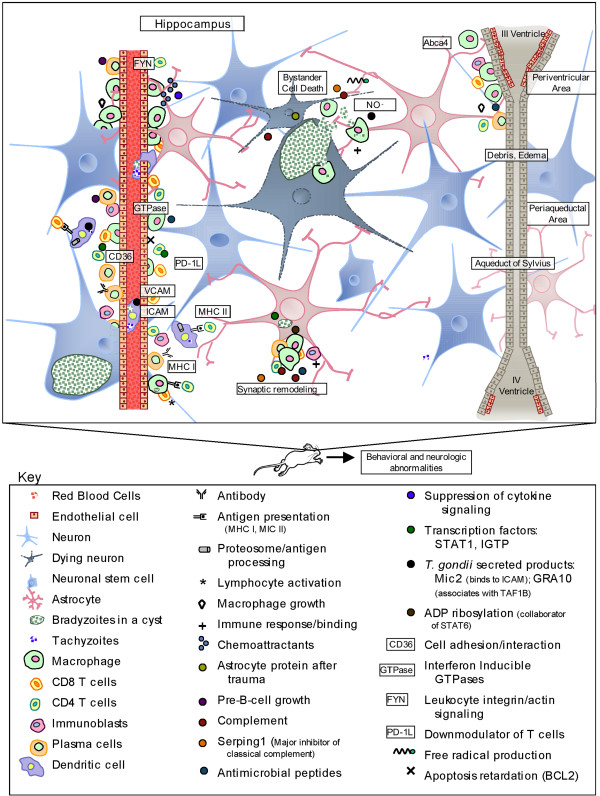
**Schematic summary of cells, molecules, and processes affected by chronic *T. gondii *infection resulting in neuronal cell loss, inflammation, and behavioral and other neurologic abnormalities in mice.** Parasites initially arrive at blood brain barrier in dendritic cells, travel into brain tissue and, within neurons, form bradyzoites in cysts. When the neuronal membrane is no longer intact, bradyzoites elicit an immune response as shown, which results in destruction of their growth and bystander cell death as shown.

Our findings, their putative pathogenic mechanisms, and consequences for neuronal cells, based on our observations herein as well as observations of others, are summarized in Figure 10. These potential pathogenic mechanisms depicted, might include usurping or interfering with functions of neurons infected by *T. gondii *bradyzoites within large cysts. The presence of bradyzoites in large cysts within synapse forming neurons has been described [101,102], but whether such synapses function normally or whether the cysts could affect neuronal function is not known. Parasites within neurons could directly result in death of infected neurons or there could be death of contiguous bystander neurons. If low-level inflammation was primed and maintained by the chronic infection, NO and toxic oxidized lipids or other toxic oxygen products could contribute to bystander cell death. Additional molecular mechanisms causing behavioral and neurologic findings in mice remain to be determined and characterized more fully. It will be of interest to determine whether some or all of these processes occur in genetically susceptible persons.

## Conclusion

In outbred mice, chronic *T. gondii *infection of a year or more duration following primary infection in early adult life causes behavioral and neurologic abnormalities and ventricular dilatation in MRIs of the brain. This reflects loss of brain parenchyma and inflammation that is particularly prominent in and contiguous to the hippocampus and around the aqueduct of Sylvius and third ventricle. There is also increased expression of message for mediators of inflammation and synaptic remodeling and markers of neuronal cell death in brains of chronically infected mice, not present in uninfected control mice. Bradyzoites may cause the inflammation when there is no neuronal membrane around cysts, which usually are sequestered in neurons. Then, these bradyzoites are engulfed by mononuclear cells leaving only rare visible free bradyzoites and virtually no free tachyzoites. These observations suggest that the ongoing inflammatory process effectively eliminates parasites outside cysts in neurons but in the process may destroy brain parenchyma. Brain weight correlates with magnitude of neurologic and behavioral abnormalities and histopathology in specific regions of the brain (including collections of plasma cells secreting antibody, T lymphocytes, macrophages in perivascular areas, microglial cells, and lymphocytes in brain parenchyma) provides the anatomic basis for the correlations. Genetic background of the host profoundly influences this process, but even in the most resistant mouse strain there is still a small amount of perivascular inflammation. Deficiency in IL4, IL13 and NRAMP are not sufficient to abrogate this inflammatory process. Microarrays provide insight into the molecular pathogenesis of the processes in the outbred mice reflecting inflammation, attenuation of this inflammation by counter regulatory processes, neuronal cell death and potential remodeling of synapses with increased expression of C1q, as well as CD36, immunoglobin genes, GFAP, and PD-1L in these transcriptomes. The implications, if any, of our findings in mice for human diseases and this very common chronic brain infection of humans remain to be determined. This mouse model of chronic *T. gondii *infection raises questions of whether persistence of this parasite in brain can cause inflammation or neurodegeneration, particularly contiguous to and in the hippocampus, periacqueductal and periventricular areas, in genetically susceptible humans.

## Competing interests

The authors declare that they have no competing interests.

## Authors' contributions

MK, EM, CW and YS maintained and infected mice and performed all other studies with mice. GH designed the general well being, behavioral and neurologic studies. GH, MK, EM and RM performed the general well being, behavioral and neurologic studies. J-HH and RPK performed and evaluated MRIs. RM and TT performed additional analyses of MRIs and identified relevant genes expressed in brain regions that were affected. GH, MK and EM harvested tissues. MK and EM isolated RNA. JWA, TM and KAK performed the full genome microarrays and the accompanying analyses. CR performed the lipid studies and analyses on sera. AWH performed special stains, including those for myelin, and analyzed these preparations. DF performed immunostaining for *T. gondii *tachyzoites and bradyzoites, cyst wall and plasma cells, and conducted the electron microscopy. FR, RW, CW, DF, YS, and RM carried out all the other microscopic analyses. LC discussed, designed and produced antibody to PD1L. KK reviewed all data and performed all statistical analyses other than for the arrays. TT and RM created the overview model for the manner in which the parasite and inflammatory process contributed to pathological process. RM, GH and JWA conceived of the study, participated in its design and coordination, and drafted the manuscript. All authors participated in the design and interpretation of data, drafted and/or revised portions of the manuscript pertinent to their own work, and have read and approved the final manuscript.

## Supplementary Material

Additional file 1 Balance and Agility of an uninfected mouse and a chronically infected mouse. Mouse number 20 is uninfected while mouse number 5 is chronically infected with *T. gondii*. Click here for file

## References

[B1] Boyer K, Marcinak J, McLeod R, Long S, Pickering LK, Prober CG (2007). *Toxoplasma gondii *(Toxoplasmosis). Principles and Practice of Pediatric Infectious Diseases.

[B2] Mortensen PB, Norgaard-Pedersen B, Waltoft BL, Sorensen TL, Hougaard D, Yolken RH (2007). Early infections of *Toxoplasma gondii *and the later development of schizophrenia. Schizophr Bull.

[B3] Flegr J, Preiss M, Klose J, Havlicek J, Vitakova M, Kodym P (2003). Decreased level of psychobiological factor novelty seeking and lower intelligence in men latently infected with the protozoan parasite *Toxoplasma gondii *Dopamine, a missing link between schizophrenia and toxoplasmosis?. Biol Psychol.

[B4] Palmer BS (2007). Meta-analysis of three case controlled studies and an ecological study into the link between cryptogenic epilepsy and chronic toxoplasmosis infection. Seizure.

[B5] Torrey EF, Bartko JJ, Lun ZR, Yolken RH (2007). Antibodies to *Toxoplasma gondii *in patients with schizophrenia: a meta-analysis. Schizophr Bull.

[B6] Derouin F, Thulliez P, Romand S (2002). Schizophrenia and serological methods for diagnosis of toxoplasmosis. Clin Infect Dis.

[B7] Hutchison WM, Bradley M, Cheyne WM, Wells BW, Hay J (1980). Behavioural abnormalities in *Toxoplasma*-infected mice. Ann Trop Med Parasitol.

[B8] Hay J, Hutchison WM, Aitken PP, Graham DI (1983). The effect of congenital and adult-acquired *Toxoplasma *infections on activity and responsiveness to novel stimulation in mice. Ann Trop Med Parasitol.

[B9] Berdoy M, Webster JP, Macdonald DW (1995). Parasite-altered behavior: is the effect of *Toxoplasma gondii *on Rattus norvegicus specific?. Parasitology.

[B10] Berdoy M, Webster JP, Macdonald DW (2000). Fatal attraction in rats infected with *Toxoplasma gondii*. Proc Biol Sci.

[B11] Webster JP (1994). The effect of *Toxoplasma gondii *and other parasites on activity levels in wild and hybrid Rattus norvegicus. Parasitology.

[B12] Webster JP (2007). The effect of *Toxoplasma gondii *on animal behavior: playing cat and mouse. Schizophr Bull.

[B13] Webster JP, Brunton CF, MacDonald DW (1994). Effect of *Toxoplasma gondii *upon neophobic behaviour in wild brown rats, Rattus norvegicus. Parasitology.

[B14] Webster JP, Lamberton PH, Donnelly CA, Torrey EF (2006). Parasites as causative agents of human affective disorders? The impact of anti-psychotic, mood-stabilizer and anti-parasite medication on *Toxoplasma gondii's *ability to alter host behaviour. Proc Biol Sci.

[B15] Vyas A, Kim SK, Giacomini N, Boothroyd JC, Sapolsky RM (2007). Behavioral changes induced by *Toxoplasma *infection of rodents are highly specific to aversion of cat odors. Proc Natl Acad Sci USA.

[B16] Webster JP (2001). Rats, cats, people and parasites: the impact of latent toxoplasmosis on behaviour. Microbes Infect.

[B17] Gelderman AH, Grimley PM, Lunde MN, Rabson AS (1968). *Toxoplasma gondii *and cytomegalovirus: mixed infection by a parasite and a virus. Science.

[B18] Grimwood BG (1985). Viral contamination of a subline of *Toxoplasma gondii *RH. Infect Immun.

[B19] Grimwood B, O'Connor G, Gaafar HA (1983). Toxofactor associated with *Toxoplasma gondii *infection is toxic and teratogenic to mice. Infect Immun.

[B20] Hibbs JB, Lambert LH, Remington JS (1971). Resistance to murine tumors conferred by chronic infection with intracellular protozoa, *Toxoplasma gondii *and Besnoitia jellisoni. J Infect Dis.

[B21] Krahenbuhl JL, Levy L, Remington JS (1974). Resistance to Mycobacterium leprae in Mice Infected with *Toxoplasma gondii *and *Besnoitia jellisoni*. Infect Immun.

[B22] Krick JA, Remington JS (1975). Resistance to infection with Nocardia asteroids. J Infect Dis.

[B23] Mahmound AA, Warren KS, Strickland GT (1976). Acquired resistance to infection with Schistosoma mansoni induced by *Toxoplasma gondii*. Nature.

[B24] McLeod R, Remington JS (1977). Studies of specificity of killing of intracellular pathogens by macrophages. Cell Immunol.

[B25] McLeod R, Cohen H, Wedderburn N, Estes R (1985). Influence of *Toxoplasma *on manifestations of Moloney virus infections. Trans Roy Soc Trop Med Hyg.

[B26] Stoicov C, Whary M, Rogers AB, Lee FS, Klucevsek K, Li H, Cai X, Saffari R, Ge Z, Khan IA, Combe C, Luster A, Fox JG, Houghton J (2004). Coinfection modulates inflammatory responses and clinical outcome of Helicobacter felis and *Toxoplasma gondii *infections. J Immunol.

[B27] Strickland GT, Voller A, Pettit LE, Fleck DG (1972). Immunodepression associated with concomitant *Toxoplasma *and malarial infections in mice. J Infect Dis.

[B28] Strickland GT, Petitt LE, Voller A (1973). Immunodepression in mice infected with *Toxoplasma gondii*. Am J Trop Med Hyg.

[B29] Strickland GT, Ahmed A, Sell KW (1975). Blastogenic response of *Toxoplasma*-infected mouse spleen cells to T- and B-cell mitogens. Clin Exp Immunol.

[B30] Strickland GT, Sayles PC (1977). Depressed antibody responses to a thymus-dependent antigen in toxoplasmosis. Infect Immun.

[B31] Welter A, Mineo JR, de Oliveira Silva DA, Lourenço EV, Vieira Ferro EA, Roque-Barreira MC, Maria da Silva N (2007). BALB/c mice resistant to *Toxoplasma gondii *infection proved to be highly susceptible when previously infected with Myocoptes musculinus fur mites. Int J Exp Pathol.

[B32] Wing EJ, Gardner ID, Ryning FW, Remington JS (1977). Dissociation of effector functions in populations of activated macrophages. Nature.

[B33] Araujo FG, Wong MM, Theis J, Remington JS (1973). Experimental *Toxoplasma gondii *infection in a nonhuman primate. Am J Trop Med Hyg.

[B34] Boulter N, Fuller S, Lees M, Jamieson SJ, de Roubaix LA, Wiley J, McLeod R, Hargrave A, Mui E, Sautter M, Remington J, Blackwell J, Smith N (2006). P2X7 susceptibility alleles in Human *Toxoplasma gondii *infection [Abstract]. IACOPA Proceedings Glasgow Scotland.

[B35] McLeod R, Cohen H, Estes R (1984). Immune response to ingested *Toxoplasma*: a mouse model of *Toxoplasma *acquired by ingestion. J Infect Dis.

[B36] Brown CR, Hunter CA, Estes RG, Beckmann E, Forman J, David C, Remington JS, McLeod R (1995). Definitive identification of a gene that confers resistance against *Toxoplasma *cyst burden and encephalitis. Immunology.

[B37] Johnson J, Suzuki Y, Mack D, Mui E, Estes R, David C, Skamene E, Forman J, McLeod R (2002). Genetic analysis of influences on survival following *Toxoplasma gondii *infection. Int J Parasitol.

[B38] Mack D, Johnson JJ, Roberts F, Roberts C, David C, Grumet C, Estes R, McLeod R (1999). HLA-Class II genes modify outcome of *Toxoplasma gondii *infection. (Rapid Communication). Int J Parasitol.

[B39] Jamieson SE, de Roubaix L-A, Cortina-Borja M, Tan HK, Mui E, Cordell HJ, Kirisits MJ, Miller EN, Peacock CS, Hargrave AC, Coyne JJ, Boyer K, Bessieres MH, Buffolano W, Ferret N, Franck J, Kieffer F, Meier P, Nowakowska DE, Malgorzata P, Peyron F, Stray-Pedersen B, Thulliez P, Wallon M, Petersen E, McLeod R, Gilbert RE, Blackwell JM (2008). Genetic and Epigenetic Factors at COL2A1 and ABCA4 influence Clinical Outcome in Congenital Toxoplasmosis. PLoS ONE.

[B40] Roberts CW, Gazzinelli RT, Khan IA, Esquivel A, Nowakowska D, McLeod R, Weiss LM, Kim K (2007). Adaptive Immunity and Genetics of the host Immune Response. Toxoplasma Gondii: The Model Apicomplexan.

[B41] Miller R, Wen X, Dunford B, Wang X, Suzuki Y (2006). Cytokine production of CD8+ immune T cells but not of CD4+ T cells from *Toxoplasma gondii*-infected mice is polarized to a type 1 response following stimulation with tachyzoite-infected macrophages. J Interferon Cytokine Res.

[B42] Saeij JP, Coller S, Boyle JP, Jerome ME, White MW, Boothroyd JC (2007). *Toxoplasma *co-opts host gene expression by injection of a polymorphic kinase homologue. Nature.

[B43] Kim SK, Fouts AE, Boothroyd JC (2007). *Toxoplasma gondii *dysregulates IFN-gamma-inducible gene expression in human fibroblasts: insights from a genome-wide transcriptional profiling. J Immunol.

[B44] Westfall AC, Lauer AK, Suhler EB, Rosenbaum JT (2005). Toxoplasmosis retinochoroiditis and elevated intraocular pressure: a retrospective study. J Glaucoma.

[B45] Bradley PJ, Ward C, Cheng SJ, Alexander DL, Coller S, Coombs GH, Dunn JD, Ferguson DJ, Sanderson SJ, Wastling JM, Boothroyd JC (2005). Proteomic analysis of rhoptry organelles reveals many novel constituents for host-parasite interactions in *Toxoplasma gondii*. J Biol Chem.

[B46] Chaussabel D, Semnani RT, McDowell MA, Sacks D, Sher A, Nutman TB (2003). Unique gene expression profiles of human macrophages and dendritic cells to phylogenetically distinct parasites. Blood.

[B47] Blader IJ, Manger ID, Boothroyd JC (2001). Microarray analysis reveals previously unknown changes in *Toxoplasma gondii*-infected human cells. J Biol Chem.

[B48] Frank O, Jones-Brando L, Leib-Mosch C, Yolken R, Seifarth W (2006). Altered transcriptional activity of human endogenous retroviruses in neuroepithelial cells after infection with *Toxoplasma gondii*. J Infect Dis.

[B49] Dong E, Guidotti A, Grayson DR, Costa E (2007). Histone hyperacetylation induces demethylation of reelin and 67-kDa glutaic acid decarboxylase promoters. Proc Natl Acad Sci USA.

[B50] Ferguson DJ, Hutchison WM, Pettersen E (1989). Tissue cyst rupture in mice chronically infected with *Toxoplasma gondii *An immunocytochemical and ultrastructural study. Parasitol Res.

[B51] Ferguson DJP, Graham DI, Hutchison WM (1991). Pathological change in the brain of mice infected with *Toxoplasma gondii*: a histological, immunocytochemical and ultrastructural study. Inter J Exp Pathol.

[B52] Oliet SH, Piet R, Poulain DA, Theodosis DT (2004). Glial modulation of synaptic transmission: Insights from the supraoptic nucleus of the hypothalamus. Glia.

[B53] Deckert-Schluter M, Albrecht S, Hof H, Wiestler OD, Schluter D (1995). Dynamics of the intracerebral and splenic cytokine mRNA production in *Toxoplasma gondii*-resistant and – susceptible congenic strains of mice. Immunology.

[B54] Remington JS, McLeod R, Thulliez P, Desmonts G, Remington JS, Klein J, Wilson CB, Baker C (2006). Toxoplasmosis. Infectious Diseases of the Fetus and Newborn Infant.

[B55] Aguzzi A, Heikenwalder M (2005). Prions, cytokines, and chemokines: a meeting in lymphoid organs. Immunity.

[B56] Lazarov O, Robinson J, Tang YP, Hairston IS, Korade-Mirnics Z, Lee VM, Hersh LB, Sapolsky RM, Mirnics K, Sisodia SS (2005). Environmental enrichment reduces Abeta levels and amyloid deposition in transgenic mice. Cell.

[B57] Monje ML, Toda H, Palmer TD (2003). Inflammatory blockade restores adult hippocampal neurogenesis. Science.

[B58] Airan RD, Meltzer LA, Roy M, Gong Y, Chen H, Deisseroth K (2007). High – Speed Imaging Reveals Neurophysiolgical Links to Behavior in an Animal Model of Depression. Science.

[B59] Andersen P, Morris R, Amaral D, Bliss T, O'Keefe J (2007). The Hippocampus Book.

[B60] McHugh TJ, Jones MW, Zuinn JJ, Balthasar N, Coppari R, Elmquist JK, Lowell BB, Fanselow MS, Wilson MA, Tonegawa S (2007). Dentate Gyrus NMDA Receptors Mediate Rapid Pattern Separation in the Hippocampal Network. Science.

[B61] Sharot T, Riccardi AM, Raio CM, Phelps EA (2007). Neural mechanisms mediating optimism bias. Nature.

[B62] University of Chicago Animal Resources Center. http://arc.bsd.uchicago.edu/.

[B63] Suzuki Y, Yang Q, Yang S, Nguyen N, Lim S, Liesenfeld O, Kojima T, Remington RS (1996). Il-4 is protective against development of toxoplasmic encephalitis. J Immunol.

[B64] Suzuki Y, Rani S, Liesenfeld O, Kojima T, Lim S, Nguyen TA, Dalrymple SA, Murray R, Remington JS (1997). Impaired resistance to the development of toxoplasmic encephalitis in interleukin-6-deficient mice. Infect Immun.

[B65] Rutschman R, Lang R, Hesse M, Ihle JN, Wynn TA, Murray PJ (2001). Cutting edge: Stat6-dependent substrate depletion regulates nitric oxide production. J Immunol.

[B66] Vidal S, Gros P, Skamene E (1995). Natural resistance to infection with intracellular parasites: molecular genetics identifies Nramp1 as the Bcg/Ity/Lsh locus. J Leukoc Biol.

[B67] Irwin S (1968). Comprehensive observational assessment: Ia A systematic, quantitative procedure for assessing the behavioral and physiologic state of the mouse. Psychopharmacologia.

[B68] Crawley JN, Paylor R (1997). A Proposed Test Battery and Constellations of Specific Behavioral Paradigms to Investigate the Behavioral Phenotypes of Transgenic and Knockout Mice. Horm Behav.

[B69] Pollack JR, Perou CM, Alizadeh AA, Eisen MB, Pergamenschikov A, Williams CF, Jeffrey SS, Botstein D, Brown PO (1999). Genome-wide analysis of DNA copy-number changes using cDNA microarrays. Nat Genet.

[B70] Bozdech Z, Llinás M, Pulliam BL, Wong ED, Zhu J, DeRisi JL (2003). The transcriptome of the intraerythrocytic developmental cycle of Plasmodium falciparum. PLoS Biol.

[B71] Hoffmann KF, Fitzpatrick JM (2004). Gene expression studies using self-fabricated parasite cDNA microarrays. Methods Mol Biol.

[B72] University of Cambridge Center for Microarray Resources. http://www.path.cam.ac.uk/resources/microarray/.

[B73] University of California, San Francisco Functional Genomics Custom Oligos. http://www.arrays.ucsf.edu/archive/meebo.html.

[B74] Illumina. http://www.illumina.com/products/dna/genomesets/meebo_mouse.ilmn.

[B75] University of Cambridge Protocols. http://www.path.cam.ac.uk/~toxo/Protocols/Protocols.html.

[B76] BlueGnome Ltd. http://www.bluegnome.co.uk/.

[B77] Microarray Image and Data Analysis (2005). R Development Core Team: A language and environment for statistical computing.

[B78] Wettenhall JM, Smyth GK (2004). limma GUI: a graphical user interface for linear modeling of microarray data. Bioinformatics.

[B79] Smyth GK, Gentleman R, Carey V, Dudoit S, Irizarry R, Huber W (2005). Linear models for microarray data. Bioinformatics and Computational Biology Solutions using R and Bioconductor.

[B80] Smyth GK, Speed TP (2003). Normalization of cDNA microarray data. Methods.

[B81] Smyth GK (2004). Linear models and Empirical Bayes Methods for Assessing Differential Expression in Microarray Experiments. Stat Appl Genet Mol Biol.

[B82] Roberts F, Roberts CW, Ferguson DJ, McLeod R (2000). Inhibition of nitric oxide production exacerbates chronic ocular toxoplasmosis. Parasite Immunol.

[B83] Ferguson DJP, Henriquez FL, Kirisits MJ, Muench SP, Prigge ST, Rice DW, Roberts CW, McLeod RL (2005). Maternal inheritance and stage specific variation of the apicoplast in *Toxoplasma gondii *during development in the intermediate and definitive host. Eukaryot Cell.

[B84] Cabana VG, Reardon CA, Wei B, Lukens JR, Getz GS (1999). SAA-only HDL formed during the acute phase response in apoAI+/+ and apoAI-/- mice. J Lipid Res.

[B85] Kang H, Liesenfeld O, Remington JS, Claflin J, Wang X, Suzuki Y (2003). TCR V beta 8+ T cells prevent development of toxoplasmic encephalitis in BALB/c mice genetically resistant to the disease. J Immunol.

[B86] Barber DL, Wherry EJ, Masopust D, Zhu B, Allison JP, Sharpe AH, Freeman GJ, Ahmed R (2006). Restoring function in exhausted CD8 T cells during chronic viral infection. Nature.

[B87] Entrez Gene. http://www.ncbi.nlm.nih.gov/entrez/query.fcgi?DB=pubmed.

[B88] GeneCards. http://www.genecards.org/.

[B89] Luft BJ, Remington JS (1992). Toxoplasmic encephalitis in AIDS. Clin Infect Dis.

[B90] McAuley J, Boyer K, Patel D, Mets M, Swisher C, Roizen N, Wolters C, Stein L, Stein M, Schey W, Remington J, Meier P, Johnson D, Heydeman P, Holfels E, Withers S, Mack D, Brown C, Patton D, McLeod R (1994). Early and longitudinal evaluations of treated infants and children and untreated historical patients with congenital toxoplasmosis: The Chicago Collaborative Treatment Trial. Clin Infect Dis.

[B91] Michel TM, Frangou S, Thiemeyer D, Camara S, Jecel J, Nara K, Brunklaus A, Zoechling R, Riederer P (2007). Evidence for oxidative stress in the frontal cortex in patients with recurrent depressive disorder – a postmortem study. Psychiatry Res.

[B92] Chiarini A, Dal Pra I, Whitfield JF, Armato U (2006). The killing of neurons by beta-amyloid peptides, prions, and pro-inflammatory cytokines. Ital J Anat Embryol.

[B93] Nam YJ, Mani K, Ashton AW, Peng CF, Krishnamurthy B, Hayakawa Y, Lee P, Korsmeyer SJ, Kitsis RN (2004). Inhibition of both the extrinsic and intrinsic death pathways through nonhomotypic death-fold interactions. Mol Cell.

[B94] Manganas LN, Zhang X, Li Y, Hazel RD, Smith SD, Wagshul ME, Henn F, Benveniste H, Djuric PM, Enikolopov G, Maletic-Savatic M (2007). Magnetic resonance spectroscopy identifies neural progenitor cells in the live human brain. Science.

[B95] Schluter D, Deckert M, Hof H, Frei K (2001). *Toxoplasma gondii *infection of neurons induces neuronal cytokine and chemokine production, but gamma interferon- and tumor necrosis factor-stimulated neurons fail to inhibit the invasion and growth of *T. gondii*. Infect Immun.

[B96] Chechneva O, Dinkel K, Cavaliere F, Martinez-Sanchez M, Reymann KG (2006). Anti-inflammatory treatment in oxygen-glucose-deprived hippocampal slice cultures is neuroprotective and associated with reduced cell proliferation and intact neurogenesis. Neurobiol Dis.

[B97] LaDu MJ, Shah JA, Reardon CA, Getz GS, Bu G, Hu J, Guo L, van Eldik LJ (2000). Apolipoprotein E receptors mediate the effects of beta-amyloid on astrocyte cultures. J Biol Chem.

[B98] Xie Z, Smith CJ, Van Eldik LJ (2004). Activated glia induce neuron death via MAP kinase signaling pathways involving JNK and p38. Glia.

[B99] Ahn HJ, Kim S, Nam HW (2007). Nucleolar translocalization of GRA10 of *Toxoplasma gondii *transfectionally expressed in HeLa cells. Korean J Parasitol.

[B100] Heikenwalder M, Zeller N, Seeger H, Prinz M, Klohn PC, Schwarz P, Ruddle NH, Weissmann C, Aguzzi A (2005). Chronic lymphocytic inflammation specifies the organ tropism of prions. Science.

[B101] Miller G (2005). Neuroscience The dark side of glia. Science.

[B102] Tran PB, Banisadr G, Ren D, Chenn A, Miller RJ (2007). Chemokine receptor expression by neural progenitor cells in neurogenic regions of mouse brain. J Comp Neurol.

[B103] Ferguson DJP, Hutchison WM (1987). An ultrastructural study of early development and tissue cyst formation of *Toxoplasma gondii *in the brains of mice. Parasitol Res.

[B104] Ferguson DJP, Hutchison WM (1987). The host-parasite relationship of *Toxoplasma gondii *in the brains of chronically infected mice. Virchows Arch A Pathol Anat Histopathol.

[B105] Ferguson DJP, Huskinson-Mark J, Araujo FG, Remington JS (1994). A morphological study of chronic cerebral toxoplasmosis in mice: comparison of four different strains of *Toxoplasma gondii*. Parasitol Res.

[B106] Ishikawa J, Kaisho T, Tomizawa H, Lee BO, Kobune Y, Inazawa J, Oritani K, Itoh M, Ochi T, Ishihara K, Hirano T (1995). Molecular cloning and chromosomal mapping of a bone marrow stromal cell surface gene, BST2, that may be involved in pre-B-cell growth. Genomics.

[B107] Ambati J, Anand A, Fernandez S, Sakurai E, Lynn BC, Kuziel WA, Rollins BJ, Ambati BK (2003). An animal model of age-related macular degeneration in senescent Ccl-2- or Ccr-2-deficient mice. Nat Med.

[B108] Miyazaki T, Dierich A, Benoist C, Mathis D (1996). Independent modes of natural killing distinguished in mice lacking Lag3. Science.

[B109] Williams MA, Bevan MJ (2006). Immunology: exhausted T cells perk up. Nature.

[B110] Deckert M, Sedgwick JD, Fischer E, Schluter D (2006). Regulation of microglial cell responses in murine *Toxoplasma *encephalitis by CD200/CD200 receptor interaction. Acta Neuropathol.

[B111] Halonen SK, Taylor GA, Weiss LM (2001). Gamma interferon-induced inhibition of *Toxoplasma gondii *in astrocytes is mediated by IGTP. Infect Immun.

[B112] Collazo CM, Yap GS, Hieny S, Caspar P, Feng CG, Taylor GA, Sher A (2002). The function of gamma interferon-inducible GTP-binding protein IGTP in host resistance to *Toxoplasma gondii *is Stat1 dependent and requires expression in both hematopoietic and nonhematopoietic cellular compartments. Infect Immun.

[B113] Trahey M, Weissman IL (1999). Cyclophilin C-associated protein: a normal secreted glycoprotein that down-modulates endotoxin and proinflammatory responses in vivo. Proc Natl Acad Sci USA.

[B114] Ockenhouse CF, Tandon NN, Magowan C, Jamieson GA, Chulay JD (1989). Identification of a platelet membrane glycoprotein as a falciparum malaria sequestration receptor. Science.

[B115] El Khoury JB, Moore KJ, Means TK, Leung J, Terada K, Toft M, Freeman M, Freeman MW, Luster AD (2003). CD36 mediates the innate host response to beta-amyloid. J Exp Med.

[B116] Triantafilou M, Gamper FG, Lepper PM, Mouratis MA, Schumann C, Harokopakis E, Schifferle RE, Hajishengallis G, Triantafilou K (2007). Lipopolysaccharides from atherosclerosis-associated bacteria antagonize TLR4, induce formation of TLR2/1/CD36 complexes in lipid rafts and trigger TLR2-induced inflammatory responses in human vascular endothelial cells. Cell Microbiol.

[B117] Eng LF, Ghirnikar RS, Lee YL (2000). Glial fibrillary acidic protein: GFAP-thirty-one years (1969–2000). Neurochem Res.

[B118] Fonseca MI, Zhou J, Botto M, Tenner AJ (2004). Absence of C1q leads to less neuropathology in transgenic mouse models of Alzheimer's disease. J Neurosci.

[B119] Stevens B, Allen NJ, Vazquez LE, Howell GR, Christopherson KS, Nouri N, Micheva KD, Mehalow AK, Huberman AD, Stafford B, Sher A, Litke AM, Lambris JD, Smith SJ, John SW, Barres BA (2007). The classical complement cascade mediates CNS synapse elimination. Cell.

[B120] Walsh T, McClellan JM, McCarthy SE, Addington AM, Pierce SB, Cooper GM, Nord AS, Kusenda M, Malhotra D, Bhandari A, Stray SM, Rippey CF, Roccanova P, Makarov V, Lakshmi B, Findling RL, Sikich L, Stromberg T, Merriman B, Gogtay N, Butler P, Eckstrand K, Noory L, Gochman P, Long R, Chen Z, Davis S, Baker C, Eichler EE, Meltzer PS, Nelson SF, Singleton AB, Lee MK, Rapoport JL, King MC, Sebat J (2008). Rare structural variants disrupt multiple genes in neurodevelopmental pathways in schizophrenia. Science.

[B121] Portugal LR, Fernandes LR, Cesar GC, Santiago HC, Oliveira DR, Silva NM, Silva AA, Lannes Vieira J, Arantes RM, Gazzinelli RT, Alvarez-Leite JI (2004). Infection with *Toxoplasma gondii *increases atherosclerotic lesions in ApoE-deficient mice. Infect Immun.

[B122] Luder CG, Lang T, Beuerle B, Gross U (1998). Down-regulation of MHC class II molecules and inability to up-regulate class I molecules in murine macrophages after infection with *Toxoplasma gondii*. Clin Exp Immunol.

[B123] Suzuki Y, Wong SY, Grumet FC, Fessel J, Montoya JG, Zolopa AR, Portmore A, Schumacher-Perdreau F, Schrappe M, Koppen S, Ruf B, Brown BW, Remington JS (1996). Evidence for genetic susceptibility to toxoplasmic encephalitis in AIDS patients. J Infect Dis.

[B124] Townsend JJ, Wolinsky JS, Baringer JR, Johnson PC (1975). Acquired toxoplasmosis. A neglected cause of treatable nervous system disease. Arch Neurol.

[B125] Couvreur J, Thulliez P (1996). Acquired toxoplasmosis of ocular or neurologic site: 49 cases. Presse Med.

[B126] Glaser CA, Honarmand S, Anderson LJ, Schnurr DP, Forghani B, Cossen CK, Schuster FL, Christie LJ, Tureen JH (2006). Beyond viruses: clinical profiles and etiologies associated with encephalitis. Clin Infect Dis.

[B127] Brooks PJ, Chen TF, Cooper L (2008). Do all of the neurologic diseases in patients with DNA repair gene mutations result from the accumulation of DNA damage?. DNA Repair (Amst).

[B128] Stetson DB, Ko JS, Heidmann T, Medzhitov R (2008). Trex1 prevents cell-intrinsic initiation of autoimmunity. Cell.

